# Identification and characterization of a putative protein disulfide isomerase (HsPDI) as an alleged effector of *Heterodera schachtii*

**DOI:** 10.1038/s41598-017-13418-9

**Published:** 2017-10-19

**Authors:** Samer S. Habash, Miroslaw Sobczak, Shahid Siddique, Boris Voigt, Abdelnaser Elashry, Florian M. W. Grundler

**Affiliations:** 1 Rheinische Friedrich-Wilhelms-University of Bonn, INRES – Molecular Phytomedicine, Karlrobert-Kreiten-Straße 13, D-53115 Bonn, Germany; 2 Department of Botany, Warsaw University of Life Sciences (SGGW), Nowoursynowska 159, PL-02787 Warsaw, Poland; 3 Rheinische Friedrich-Wilhelms-University of Bonn, Department of Plant Cell Biology, Institute of Cellular and Molecular Botany, Kirschallee 1, D-53115 Bonn, Germany; 4 Strube Research GmbH & Co. KG, Hauptstraße 1, 38387 Söllingen, Germany

## Abstract

The plant-parasitic nematode *Heterodera schachtii* is an obligate biotroph that induces syncytial feeding sites in roots of its hosts. Nematodes produce effectors that are secreted into the host and facilitate infection process. Here we identified *H. schachtii* protein disulphide isomerase (HsPDI) as a putative effector that interferes with the host’s redox status. *In situ* hybridization showed that *HsPdi* is specifically localized within esophageal glands of pre-parasitic second stage juveniles (J2). *HsPdi* is up-regulated in the early parasitic J2s. Silencing of *HsPdi* by RNA interference in the J2s hampers their development and leads to structural malfunctions in associated feeding sites induced in Arabidopsis roots. Expression of HsPDI in Arabidopsis increases plant’s susceptibility towards *H. schachtii*. *HsPdi* expression is up-regulated in the presence of exogenous H_2_O_2_, whereas *HsPdi* silencing results in increased mortality under H_2_O_2_ stress. Stable expression of HsPDI in Arabidopsis plants decreases ROS burst induced by flg22. Transiently expressed HsPDI in *N. benthamiana* leaves is localized in the apoplast. HsPDI plays an important role in the interaction between nematode and plant, probably through inducing local changes in the redox status of infected host tissue. It also contributes to protect the nematode from exogenous H_2_O_2_ stress.

## Introduction

Plant-parasitic cyst nematodes are obligate biotrophs that induce and maintain intimate and long-term feeding relationships with their host plants. Second stage juveniles (J2s) of cyst nematodes hatch from eggs and invade the roots primarily in the elongation zone^1^. After entering the roots, nematodes pierce single cells with their stylet, penetrate them and migrate through various tissue layers until they reach the vascular cylinder^1^. Nematode migration inside the root is aided by releasing cell wall-degrading enzymes via the mouth stylet. Reaching the vascular cylinder, J2s select a suitable cell to establish an initial syncytial cell (ISC). Once an ISC is established, cell walls of neighbouring cells are partially dissolved and the protoplasts of these cells fuse^2–4^. This process continues so that a multinucleate, hypertrophied and metabolically hyperactive syncytium is formed. Syncytium formation is accompanied by massive transcriptomic and metabolomics changes, which have been previously reported^5,6^. After establishment of the ISC, J2s pursue their life cycle, increase in size and moult three times (J3, J4, and adult) until reaching adult stages. Adult males leave the roots to search for females to mate, whereas the lemon-shaped adult females remain attached to the roots. After mating, females lay eggs inside their bodies, then die and turn into cysts protecting the eggs from the surrounding hostile environment^1,3^. For successful parasitism, *H. schachtii* releases various effectors into the plant that help the nematodes successfully invade the roots, suppress the plant’s defence mechanisms, induce and maintain the syncytium^7–9^. The nematode effectors 4F01 and 10A06 are examples of effectors manipulating plant defence. 4F01 mimicks plant annexin and thereby alters host defence against nematodes^10^. The host’s ability to produce defense-associated compounds such as salicylic acid also is disturbed by the interaction between the effector 10A06 and plant spermidine synthase^11^. In addition, nematodes are also able to produce molecules with hormone activity. These effectors enhance the plant’s activities to the benefit of the nematodes. Recently, it was shown that cell division and growth were stimulated for feeding site formation by secreting cytokinins from *H. schachtii* J2s into the feeding site^12^. Additionally, nematodes secrete peptides, which mimic CLE plant peptide hormones and reprogram the root cells in order to initiate and maintain feeding sites^13^.

Plants are hosts to a wide range of pathogens, including bacteria, fungi, viruses, insects and nematodes. During evolution, both pathogens and plants have developed various strategies to facilitate their efforts, resembling a continuous battle of actions and counteractions^9–17^. One of the responses by which plants defend themselves against pathogens is the production of reactive oxygen species (ROS). Because ROS are highly toxic and reactive, they can restrict pathogen growth and development. In addition to their role in plant defence, ROS have also been shown to act as signalling molecules and regulate a variety of key biological processes, such as growth, differentiation, proliferation, and apoptosis^18–21^. A number of studies have demonstrated and clarified the positive role of ROS in environmental stresses other than plant defence. ROS with prominent biological significance include superoxide anion (O^−2^), hydroxyl radical (OH-) and hydrogen peroxide (H_2_O_2_). Among these, H_2_O_2_ is less reactive and can freely diffuse through lipid membrane, thus making it an ideal candidate for signalling processes. A number of studies have shown the correlation between ROS levels and intensity of pathogen infections^14,22^. In the context of plant-nematode interactions, the presence of ROS-generated signals and their spatiotemporal expression in the interaction of tomato with root-knot nematodes have been studied in detail^23^. In Arabidopsis, invasion and parasitism by *H. glycines* were shown to induce H_2_O_2_ production not only in the infected cells but also in the cells which are not in direct contact with nematodes^24^. Similarly, it has recently been shown that infection of Arabidopsis *by H. schachtii* activates the plasma membrane-localised NADPH oxidase (RbohD and RbohF) to produce ROS, which, however, is required for proper infection and syncytium development^25^. These observations on parasitic nematodes together with other previously published literature led to the suggestion that redox homeostasis is crucial for both effective plant defence and successful parasitism^25,26^.

Because ROS are highly toxic, the development of an efficient scavenging system is crucial for both plants and pathogens. This is especially important in case of biotrophic pathogens, who require living cells for successful infection^27–32^. Animal parasitic nematodes have developed several ROS-scavenging mechanisms to protect themselves against the oxidative defence mechanisms of their hosts^33,34^. Two animal parasitic nematodes species, *Brugia malayi* and *Haemonchus contortus*, possess different thioredoxins that have been shown to increase nematode immunity against host ROS production^34,35^.

Plant parasitic nematodes have also been shown to be capable of manipulating the redox status of their hosts^36^. The root-knot nematode *Meloidogyne incognita* secretes peroxiredoxins to successfully develop within its tomato host^31^. *Meloidogyne javanica* produces a transthyretin-like protein, MjTTL5, which has been shown to manipulate the host immune system by interacting with the Arabidopsis ferredoxin-thioredoxin reductase catalytic subunit (AtFTRc), that plays an important role in the ferredoxin/thioredoxin regulatory chain and decrease ROS burst^36^. The potato cyst nematode (*Globodera rostochiensis*) produces peroxiredoxins (PXN) and glutathione peroxidases (GXP), which are likely responsible for regulation of ROS level at nematode infection sites^27,37^.

Protein disulfide isomerase (PDI) family includes PDI and PDI-like proteins with thioredoxin domains, also called thioredoxin superfamily. They vary in size, expression, localization and enzymatic function. Typical PDI consists of four thioredoxin-like domains, the domains (a and a′) with catalytic domains are separated by two non-catalytic domains (b and b´). In addition to this, an ER retention signal is located at the small C-terminal domain (c), and a signal peptide at the N-terminus. The two catalytic domains containing characteristic CGHC active-site motif are essential for PDI enzymatic activity^38^. PDIs are very versatile enzymes as they are able to catalyze *in vitro* thiol oxidation reactions and disulfide reduction or isomerisation, depending on their redox states^39^. PDIs that are found in an oxidized form most likely function as thiol oxidases, whereas PDIs functioning as isomerases need to be in a reduced state^40^. In addition, it was found that PDI plays an important role in the regulation of ROS. Oxidized PDI stimulated ROS production whereas reduced PDI inhibited the production of ROS^41^.

PDIs play an important role during host-pathogen interactions^42^. It was shown that the expression of *Leishmania* PDI (LmPDI) is higher in virulent parasitic strains of *Leishmania*, suggesting that PDI protein is a virulence factor^43^. The use of PDI inhibitors affected parasite growth^44^. Studying the expression pattern of the PfPDI-8 from *Plasmodium falciparum* showed that it is associated with all parasitic stages^45^. Recently, a PDI from the oomycete plant parasitic *Phytophthora parasitica* (PpPDI1) was identified as a virulence factor. It was shown that expressing PpPDI1 induce strong cell death in *Nicotiana benthamiana* leaves while mutating the gene decreased the effect^46^. To date, nothing is known about the role played by PDIs in *H. schachtii*. Here, we describe a effector candidate of *H. schachtii* which belongs to the PDI family. We show that it is involved in the interaction with the nematode’s host plant and in protecting the parasite against plant-released ROS.

## Results

### Sequence analysis of HsPDI

**Figure 1:**
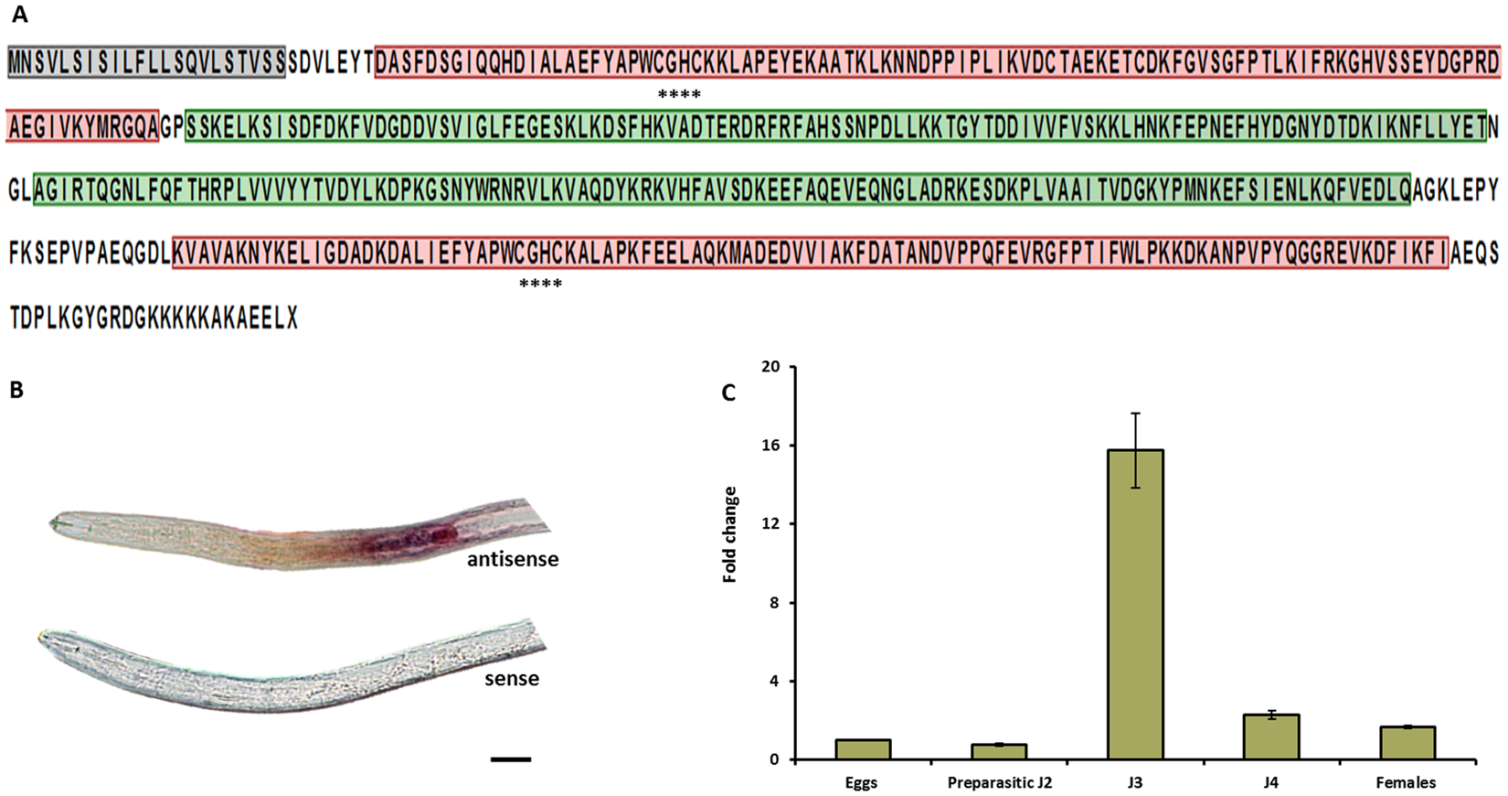
Structure and functional annotation of HsPDI and expression pattern of *HsPdi* gene. (**A**) Detailed amino acid sequence of HsPDI protein with predicted signal peptide (grey), thioredoxin domains (a and a’ in red, b and b’ in green) and functional catalytic active site (asterisks). (**B**) *In situ* hybridization of DIG-labelled antisense *HsPdi* probe to pre-parasitic J2s showed transcripts localized inside the sub-ventral oesophageal gland (purple stain). The sense probe was used as control. Bars: 20 μm. (**C**) The relative expression levels of *HsPdi* mRNA quantified using qRT-PCR. The fold change values of changes in *HsPdi* mRNA abundance in pre-parasitic J2, J3, J4, and female relative its abundancy in eggs. Data are averages of three biologically independent experiments, each consisting of three technical replicates. *H. schachtii* actin was used as an internal control to normalize gene expression level.

A partial sequence of the *Heterodera glycines* esophageal gland cells putative secretory protein hsp3 (AF273730.1) was used to identify a homologue in *H. schachtii*. BLASTn results revealed 100% similarity of hsp3 (AF273730.1) with one of the contigs in our draft transcriptome. The contig was extracted and further analysed. A detailed sequence analysis showed that the resulted contig contains a signal peptide of 22 amino acid residues and lacks a transmembrane domain. Further domain analysis with NCBI CD search program revealed four conserved thioredoxindomains (a,b,b’,a’) with two catalytic domains (CGHC), as outlined in Fig. 1A. This arrangement of the domains is a classical feature of the protein disulfide isomerase family members^47^. For that reason, the described sequence was annotated as HsPDI.

Alignment results of HsPDI with other PDIs from different organisms showed high conserved active domains (CGHC) in eukaryotic organisms including some protozoans (Supplementary Fig. S1). However, overall the highest level of sequence similarity among PDIs was within nematodes (identity >70%). In comparison, similarity was less to the other organisms PDIs (Supplementary Table S1).

### *HsPdi* is expressed in oesophageal glands during early parasitic stages

We used *in situ* hybridization to localise the expression of the *HsPdi* in the pre-parasitic juveniles of *H. schachtii*. The labelled antisense riboprobe of *HsPdi* gave a clear signal in the sub-ventral gland cell of the pre-parasitic J2s while no signal was observed in the labelled sense (negative control) (Fig. 1B). To further investigate the expression pattern of *HsPdi* during different developmental stages of *H. schachtii*, we used the qRT-PCR using cDNA generated from nematode RNA isolated at different pre-parasitic (eggs and freshly hatched J2s) and parasitic developmental stages (J3s, J4s and young females). *HsPdi* expression level increased during the sedentary stages of nematode development, reaching its maximum in J3 with 15-fold increase compared with unhatched J2 in eggs. In J4s and young females, expression decreased, respectively, to 2.3 and 1.6-fold compared with unhatched J2s in eggs and hatched pre-parasitic J2s (Fig. 1C).

### HsPDI is involved in parasitism

**Figure 2:**
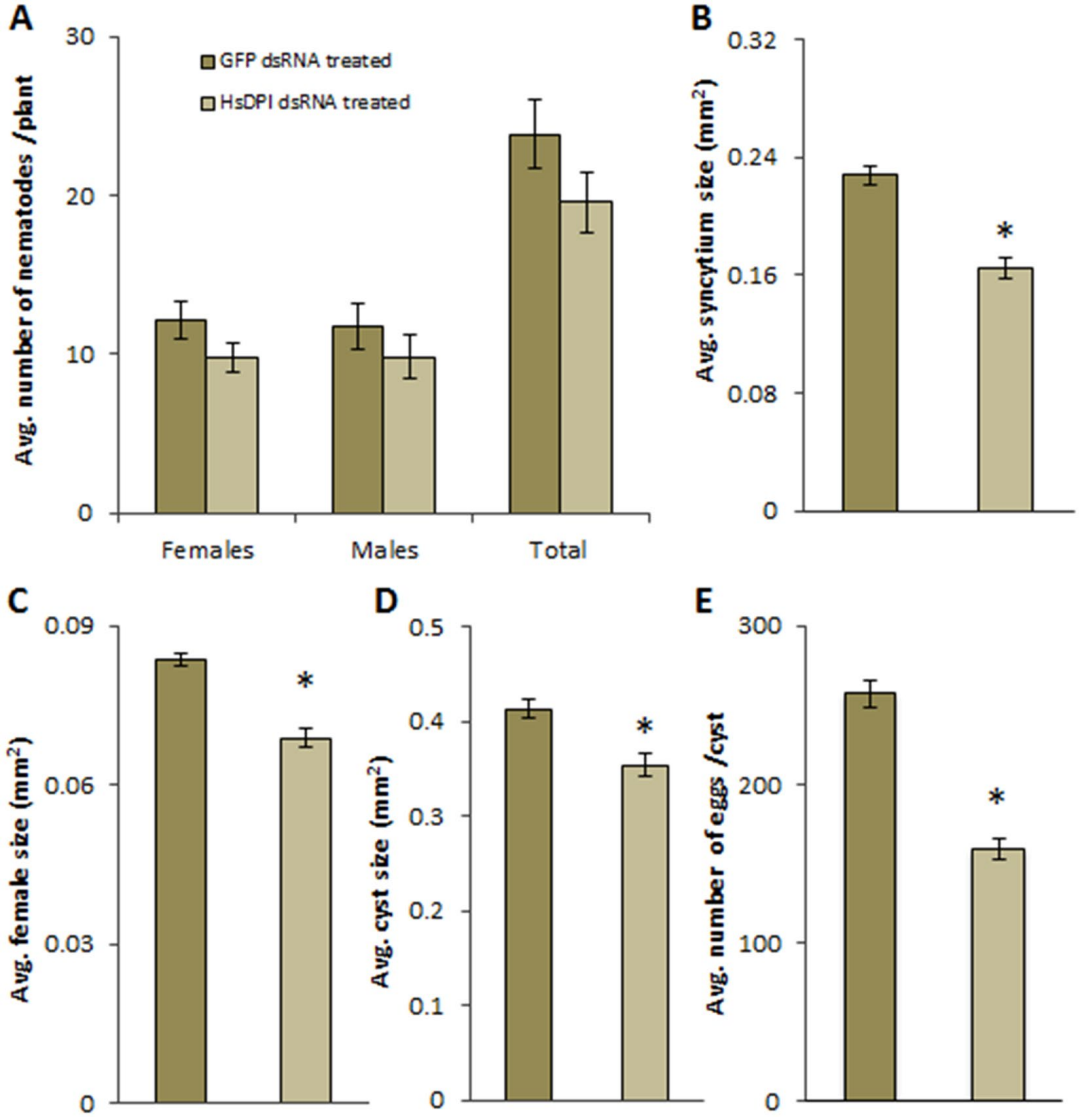
Effect of *HsPdi* silencing on *H. schachtii* parasitism. Figures show susceptibility parameters representing the parasitism of the nematodes that were soaked in the *HsPdi* dsRNA compared with those nematodes which were soaked in *GFP* dsRNA as a negative control (**A**) Average number of males, females and total nematodes developed on Arabidopsis Col-0 plant. (**B**) Average sizes of syncytia at 13 DAI (**C**) Average sizes of females at 13 DAI. (**D**) Average sizes of cysts at 45 DAI. (**E**) Average numbers of eggs inside cysts. Data are based on three independent experiments. Each bar represents the mean ± standard error of n > 35. Asterisk marks indicate significant differences based on Student’s *t*-test (P < 0.05).

To analyse whether *HsPdi* plays a role in parasitism, we performed *in vitro* RNAi targeting *HsPdi* (see methods for details). Our results showed that RNAi caused a significant decrease in the transcript abundance of *HsPdi* in J2s of *H. schachtii* (Supplementary Fig. S2). Next we infected the roots of Arabidopsis wild-type plants with nematodes soaked in dsRNA targeted against *HsPdi* or *GFP* and counted the number of females and males at 12 DAI. We also measured the average sizes of females, associated syncytia and cysts and counted the average number of eggs per cyst. Our analysis showed no significant difference in number of infecting nematodes found on plants infected with J2s treated with dsRNA targeting *HsPdi* compared to those treated with dsRNA targeting *GFP* (Fig. 2A). However, the average size of syncytia, average size of females, average size of cysts and average number of eggs per cyst were reduced significantly in plants infected with juveniles treated with dsRNA against *HsPdi* compared with *GFP* (Fig. 2B–E).

**Figure 3:**
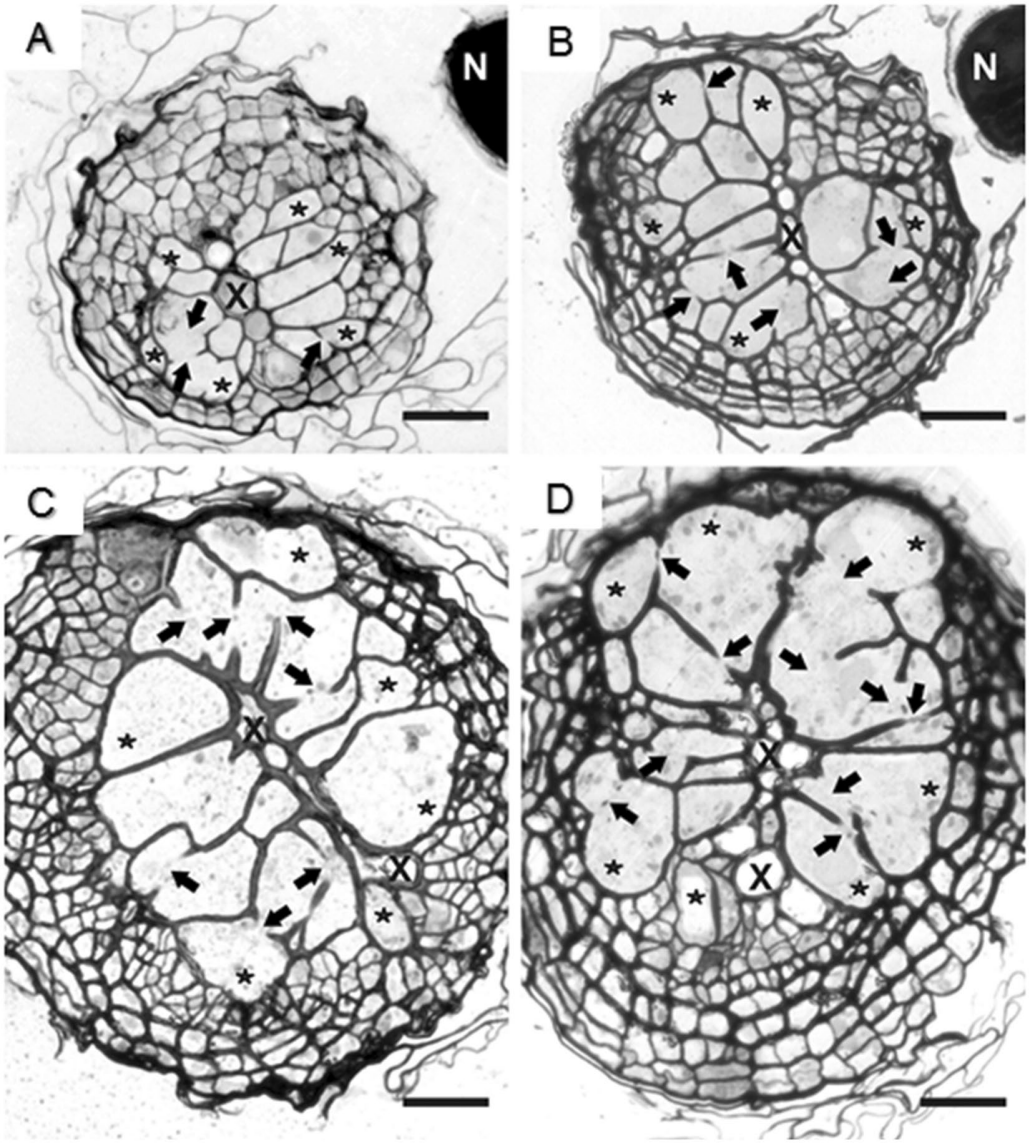
Anatomy of nematode-induced syncytia. Light microscopy images of cross sections of syncytia at (**A**,**B**) 5 DAI and (**C**,**D**) 10 DAI induced in Arabidopsis Col-0 roots upon infection with J2s treated with *HsPdi* dsRNA (**A**,**C**) or *GFP* dsRNA (**B**,**D**). Selected syncytial elements are marked with asterisks and cell wall openings are pointed with arrows. Abbreviations: N, nematode; X, xylem. Bars: 20 µm.

To characterise further these differences, we examined the anatomical and ultrastructural features of syncytia induced by *HsPdi* or *GFP* dsRNA-treated juveniles, and performed a detailed time-course analysis via light and transmission electron microscopy. To obtain comparable materials for these analyses, we took sections in the middle region of syncytia. At 5 DAI, syncytia induced by *HsPdi* dsRNA-treated juveniles were composed of a similar number of elements as syncytia induced by the *GFP* dsRNA-treated control juveniles (Fig. 3A and B). Nevertheless, the hypertrophy of a single syncytial element was lower with *HsPdi* silenced nematodes. The openings were also observed to be narrower than those found in syncytia induced by *GFP* dsRNA-treated juveniles. At 10 DAI, the differences between syncytia induced by *HsPdi* and *GFP* dsRNA-treated juveniles were less obvious (Fig. 3C and D), but the number and extent of cell wall openings were lower and the regions with confluent cytoplasm were smaller in syncytia induced by *HsPdi* dsRNA-treated juveniles.

**Figure 4:**
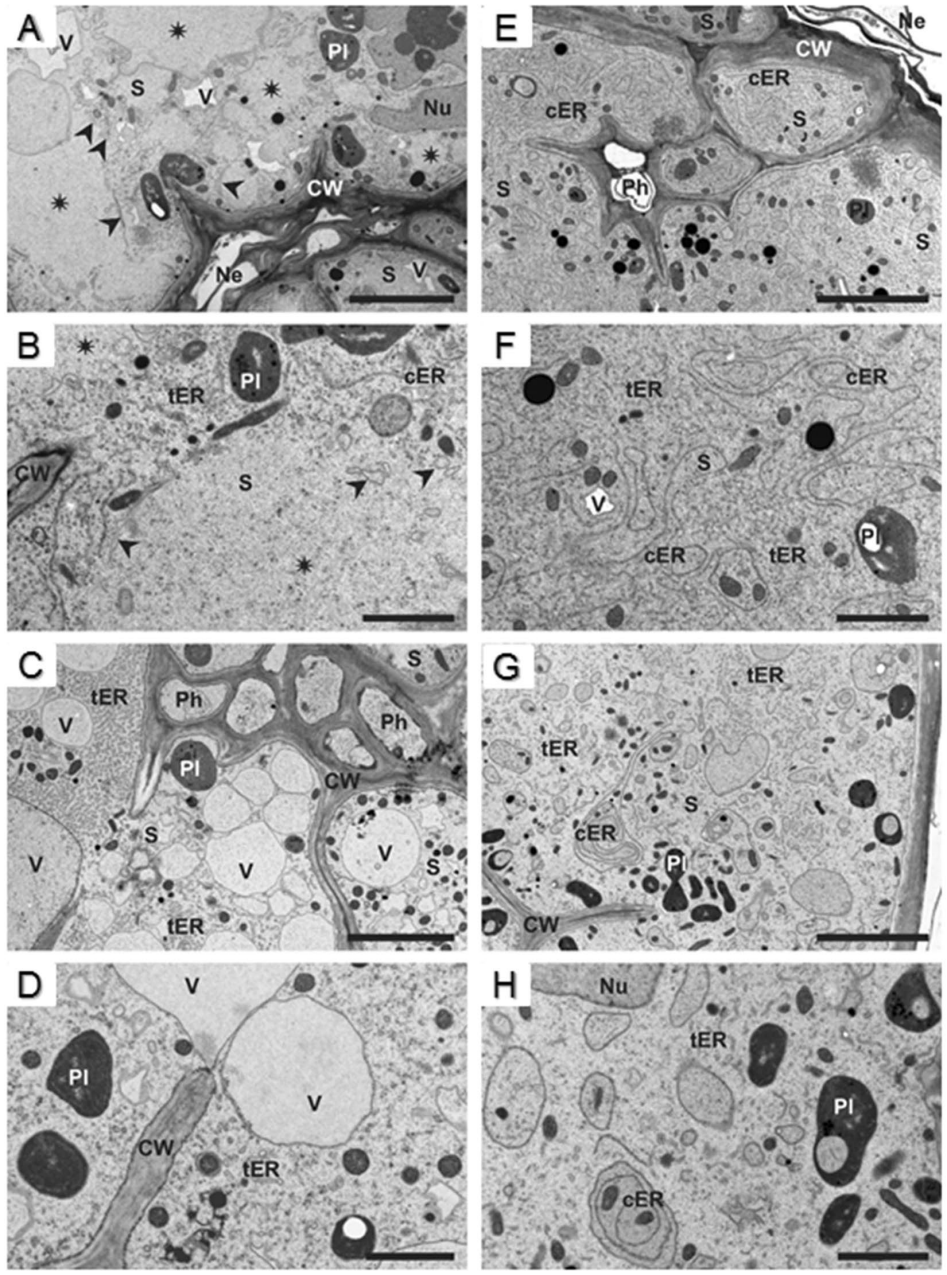
Ultrastructure of nematode-induced syncytia. Transmission electron microscopy images of cross sections of syncytia at 5 (**A**,**B**,**E**,**F**) and 10 DAI (**C**,**D**,**G**,**H**) induced in Arabidopsis Col-0 roots upon infection with J2s treated with *HsPdi* dsRNA (**A–D**) or *GFP* dsRNA (**E–H**). Asterisks indicate organelle-free regions in syncytial cytoplasm, arrow-heads point to dilated cisternae at the interface of organelle-free region and regular syncytial cytoplasm. Abbreviations: CW, cell wall; cER, cisternal endoplasmic reticulum; tER, tubular endoplasmic reticulum; Ne, necrosis; Nu, nucleus; Ph, phloem; Pl, plastid; S, syncytium; V, vacuole/vesicle. Bars: 5 µm (**A**,**C**,**E**,**G**) and 2 µm (**B**,**D**,**F**,**H**).

Parallel examinations of the ultrastructure of syncytial elements revealed developmental abnormalities in syncytia induced by *HsPdi* dsRNA-treated juveniles. They showed differences in the electron density of the cytoplasm, the organisation and composition of endoplasmic reticulum (ER) and vacuole formation. First of all, cytoplasm electron density was lower in syncytia induced by *HsPdi* dsRNA-treated juveniles at 5 DAI (syncytia associated with sedentary J2) and 10 DAI (syncytia associated with young females) than in syncytia induced by *GFP* dsRNA-treated juveniles (Fig. 4A,B,C vs E,F). However, this difference was less pronounced when comparing syncytia at 10 DAI (Fig. 4C,D vs. G,H). In addition, large organelle-free regions were present in syncytia induced by *HsPdi* dsRNA-treated juveniles at 5 DAI (Fig. 4A,B). At the interface to the organelle-containing cytoplasm, small vesicles or dilated cisterns of accumulated ER appeared (Fig. 4A,B). Besides, the organization and composition of the ER differed strongly between syncytia induced by both groups of juveniles. In control syncytia, numerous cisternae of ER were present (Fig. 4F), whereas they were almost absent in syncytia induced by *HsPdi* dsRNA-treated juveniles at 5 DAI (Fig. 4C). Interestingly the total number of ER cisternae decreased during syncytia development in both groups. At 10 DAI they were still quite numerous in syncytia induced by *GFP* dsRNA-treated juveniles (Fig. 4G,H), but completely absent in syncytia induced by *HsPdi* dsRNA-treated juveniles (Fig. 4C,D). The ER system in syncytia induced by *HsPdi* dsRNA-treated juveniles consisted predominantly of tubular ER that occupied large regions of syncytial cytoplasm (Fig. 4A,C). Tubular ER was present also in syncytia induced by *GFP* dsRNA-treated juveniles, but never appeared alone in any syncytial element (Fig. 4E–H). Thirdly, syncytia induced by *HsPdi* dsRNA-treated juveniles were strongly vacuolated at 10 DAI (Fig. 4C,D). Organelles such as nuclei, plastids or cell walls, displayed no structural changes (Fig. 4A–H), and their ultrastructure was typical as described for syncytial elements^4,48^.

**Figure 5:**
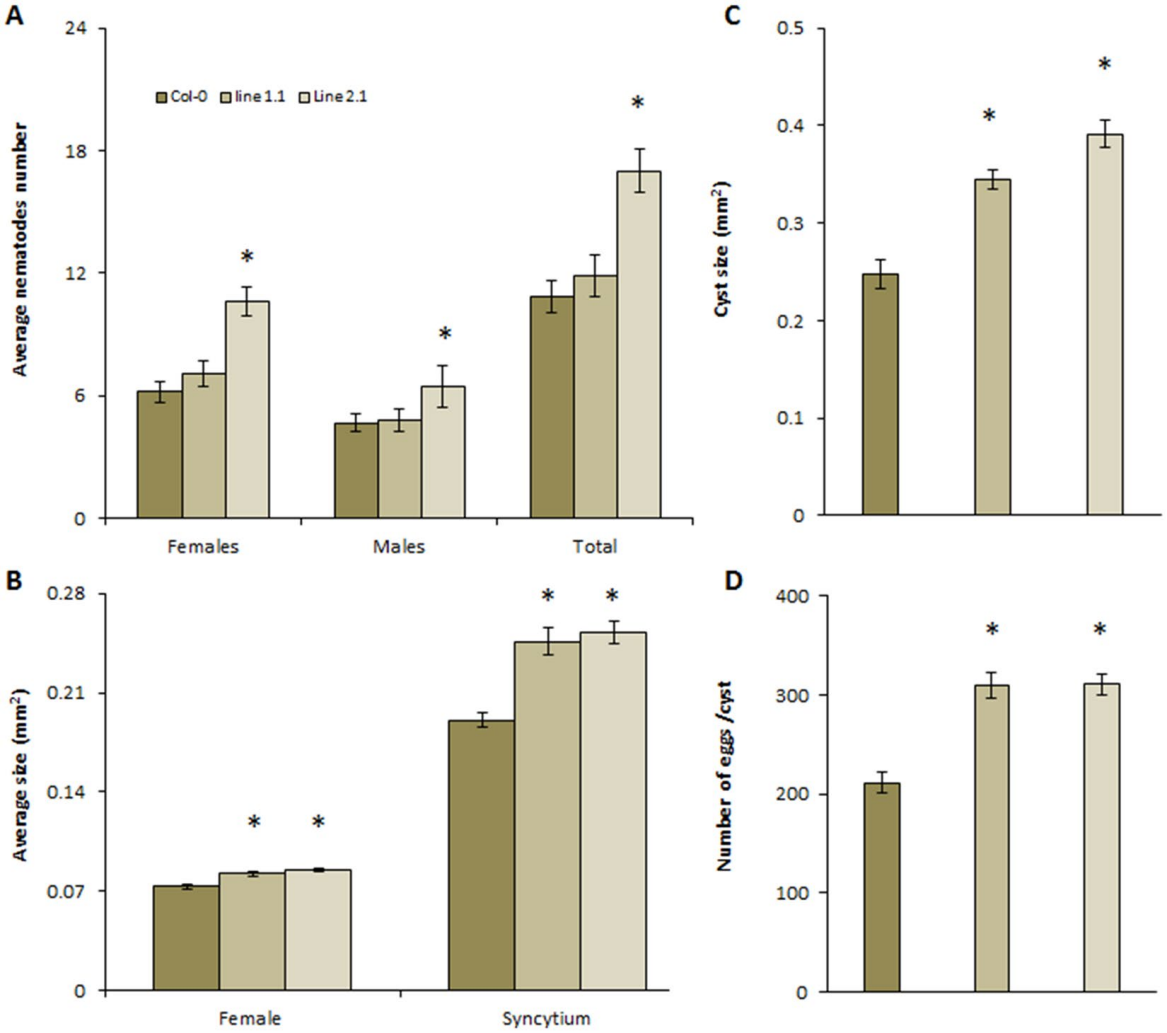
Effect of HsPDI expression on Arabidopsis susceptibility to *H. schachtii* infection. Stable transformed Arabidopsis plants expressing HsPDI gene (Line 1.1 and 2.1) were infected with J2s of *H. schachtii*. Susceptibility parameters were. (**A**) Average numbers of females, males and total nematodes per plant. (**B**) Average sizes of females and syncytia at 13DAI. (**C**) Average sizes of cysts at 45 DAI. (**D**) Average numbers of eggs inside cysts. Data are based on three independent experiments. Each bar represents the mean ± standard error of n > 35. Asterisk marks indicates significant differences based on Student’s *t*-test (P < 0.05).

To analyse the function of *HsPdi* in more detail, we produced transgenic Arabidopsis plants over-expressing HsPDI (35S::*HsPdi*-GFP). The relative expression of *HsPdi* in transgenic lines was measured using qRT-PCR (Supplementary Fig. S3 **)**. A detailed phenotypic analysis did not reveal any significant differences in plant growth as indicated by number of lateral roots, length of main root, fresh root weight and fresh shoot weight between HsPDI expressing plants and Col-0 (Supplementary Fig. S4). Next, we analysed these lines for susceptibility via nematode infection assay and found that at least one of the HsPDI expressing lines was more susceptible to *H. schachtii* infection as compared with wild-type control (Fig. 5A). However, considering significantly higher average female, cyst, and syncytium sizes, both HsPDI expressing lines appear to be more susceptible than the wild type Col-0 plants (Fig. 5B,C). Furthermore, the average number of eggs were also increased significantly compared with the control (Fig. 5D).

### *HsPdi* expression is triggered by H_2_O_2_ and increases H_2_O_2_ tolerance

**Figure 6:**
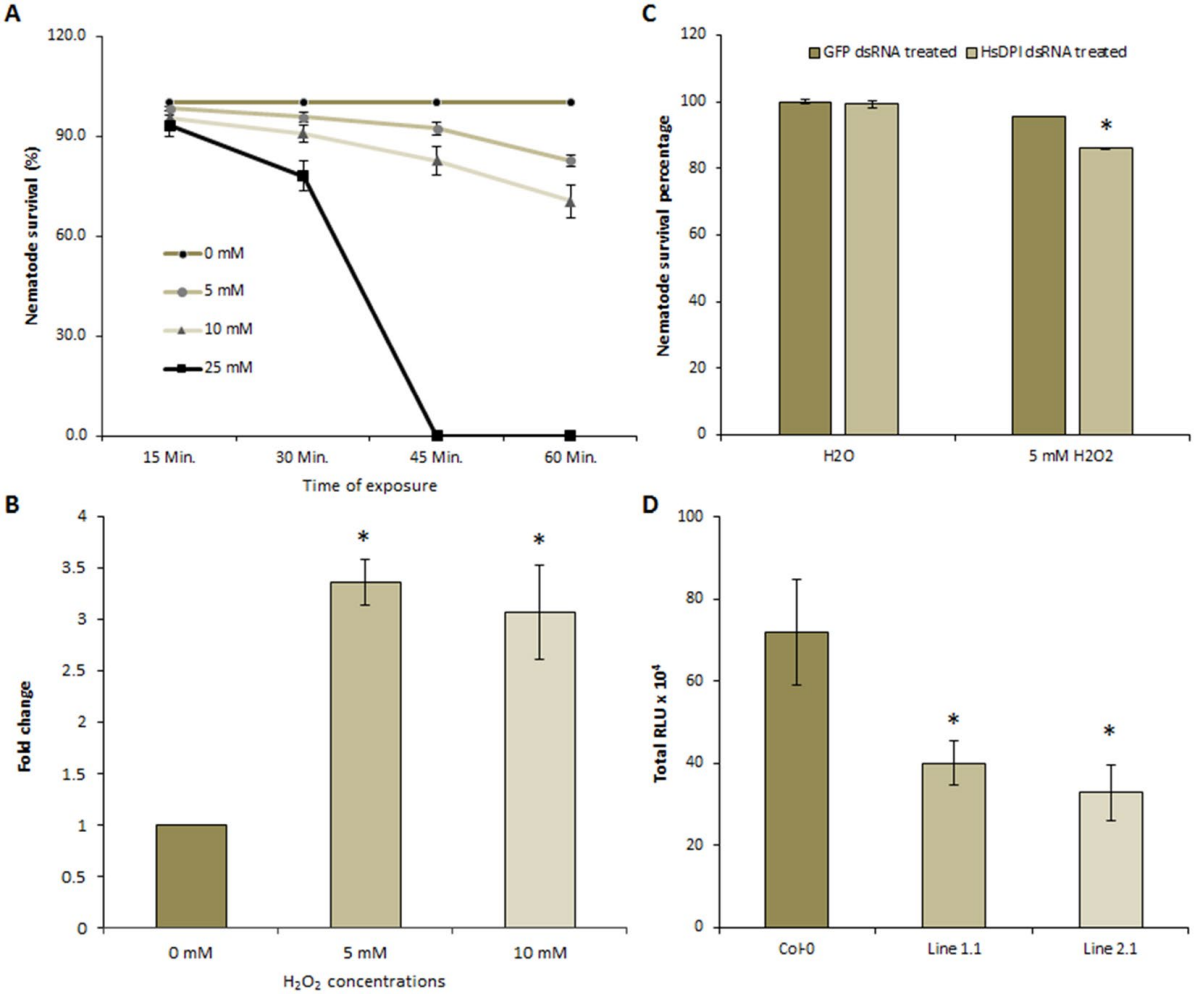
HsPDI expression is triggered by H_2_O_2_ and increases H_2_O_2_ tolerance. (**A**) Mortality rate of freshly-hatched J2s in H_2_O_2,_ n = 9. (**B**) The relative *HsPdi* mRNA expression levels in freshly hatched J2s quantified using qRT-PCR after soaking for 30 min in 5 and 10 mM H_2_O_2_. The fold change values were calculated in H_2_O_2_ incubated nematodes relative to J2s soaked in sterile distilled water (0 mM H_2_O_2_), n = 9. (**C**) Effect of *HsPdi* silencing on H_2_O_2_ stress tolerance, freshly hatched J2s of *H. schachtii* were soaked in *HsPdi* dsRNA or *GFP* dsRNA as a control. dsRNA-treated nematodes were soaked in 5 mM H_2_O_2_ or in sterile water and alive nematodes were counted after 30 min, n = 12. (**D**) ROS bursts in response to the bacterial elicitor peptide flg22 was measured in relative light units (RLU) in plants expressing HsPDI and compared with Col-0 using luminol-based assay after 120 min-long incubation, n = 12. Data are based on three independent experiments. Each bar represents the mean ± standard error. Asterisk marks indicates significant differences based on Student’s *t*-test (P < 0.05).

We tested whether treating J2s with H_2_O_2_ influence the survival of nematodes. To test vitality, pre-parasitic J2s were soaked in 5, 10 or 25 mM H_2_O_2_ and the percentage of dead juveniles was evaluated after 15, 30, 45, and 60 min. We found that J2s can survive up to 30 min in 10 mM H_2_O_2_ without significant increase in mortality rate (Fig. 6A), but prolonged treatment substantially increased mortality of J2s. Next, we used qRT-PCR to analyse the expression of *HsPdi* in response to H_2_O_2._ By analysing the juveniles that were exposed to H_2_O_2_ (5 or 10 mM) for 30 min, we found a significant increase in transcript abundance of the *HsPdi* when compared with water-treated control J2s (Fig. 6B).

We examined the mortality rate of *HsPdi* or *GFP* dsRNA-treated J2s after soaking them in 5 mM H_2_O_2_ for 30 min. As a control, we incubated dsRNA-treated J2s in water **(**Fig. 6C). We observed a significantly lower percentage of J2s that survived in H_2_O_2_ in case of *HsPdi* dsRNA-treated nematodes, indicating that *HsPdi* plays a role in protecting J2 from the impact of the exogenous H_2_O_2_. Following up on these results, we investigated whether HsPDI could modulate the plant endogenous ROS burst. We incubated the plants leaf discs in presence of the bacterial peptide flg22 and total ROS burst was measured in HsPDI expressing transgenic plants and wild type Col-0. We observed a significant decrease in total ROS in both transgenic plant lines expressing the HsPDI to half of the total amount of ROS produced by Col-0 plants (Fig. 6D, Supplementary Fig. S5), while no significant changes were found in the transgenic plant lines expressing Hs-Tyr compared with Col-0 (Supplementary Fig. S6).

### HsPDI is localised in the plant apoplastic space

**Figure 7:**
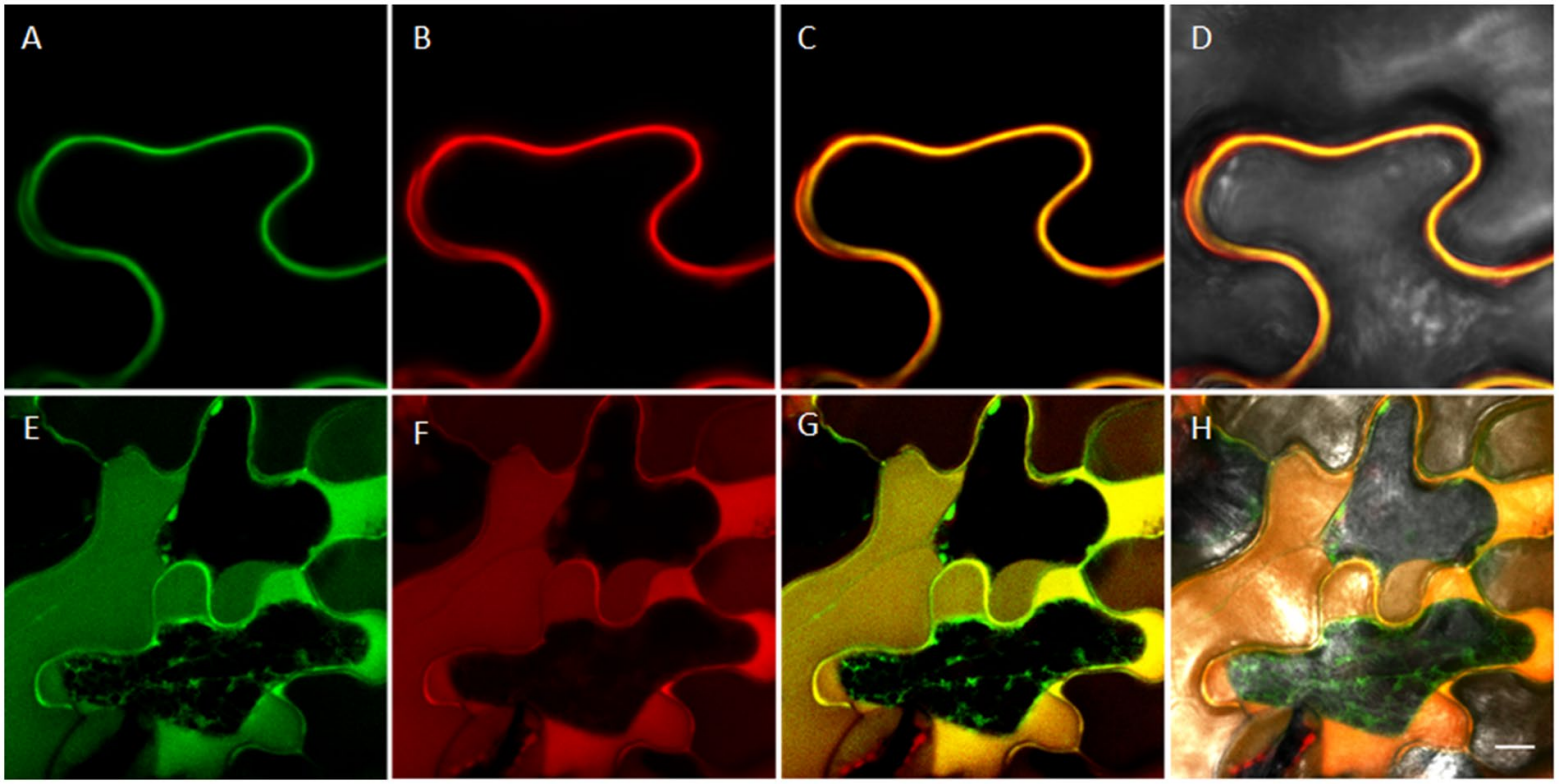
Subcellular localization of HsPDI:GFP within *Nicotiana benthamiana* leaf epidermal cell. Observation was done at 6 days post inoculation with Agrobacterium infiltrated at OD600nm = 1 **(A–D)** Florescent signal in the outer cell periphery. **(E–H)** The signal localized in the outer cell periphery after plasmolysis in 1 M NaCl for 10 min. (**A,E**) Green fluorescence originated from HsPDI:GFP fusion protein. **(B,F)** Red fluorescence originated from apoplastic marker::mCherry fusion protein. **(C,G)** Merged image shows the red and the green signal in orange. (**D**,**H)** Merged image shows the florescent signal in bright field. Bars = 10 μm.

To determine the sub-cellular localization of the HsPDI protein inside the host cells, we transiently expressed HsPDI*::GFP* in *Nicotiana benthamiana* epidermal cells with the constitutive cauliflower mosaic virus (CaMV) 35S promoter and assessed its localization by co-infiltrate it with an apoplastic marker under the confocal microscope (Fig. 7A–D). Our observations clearly showed that GFP signal was localized in the cell periphery and co-localized with the mCherry signal. To further investigate the specificity of subcellular localization, we induced plasmolysis by adding 1 M NaCl to the leaf tissue. Upon dissociation of plasma membrane from the cell wall, the signal was observed in the apoplastic space indicating the localization of HsPDI in outer cell periphery (Fig. 7E–H).

## Discussion

Plant endo-parasitic nematodes spend most of their life cycle inside host tissue. To establish parasitism, nematodes have evolved a repertoire of physical and chemical tools including secretions of proteinaceous and non-proteinaceous effectors into the host tissues^8,49^ In the current work, we identified from *H. schachtii* draft transcriptome a novel putative effector gene (*HsPdi*) encoding a protein with a protein disulfide isomerase domain. We demonstrated its importance in nematode parasitism and ROS detoxification.

HsPDI encodes a 22-amino acid, signal peptide at its N-terminal and lacks a transmembrane domain. Moreover, four thioredoxin domains and two active catalytic motifs were also detected (CGHC and CGHC) (Fig. 1A). Being a typical PDI protein, HsPDI contains the main structural building block. It also contains a-type domains containing two cysteines in a CXXC active-site motif with an intervening GH sequence, which is the most common CGHC motif in the PDIs^47^. Presence of a signal peptide and lack of a transmembrane domain supported the role of HsPDI as a putative effector^50,51^. Previous studies have shown that the active motifs are essential for the protein function as mutations in the active site domains result in the loss of protein functions^52^. It was also shown that, as a virulent factor, mutating the active motifs of the PpPDI1 abolished necrosis-inducing activity of the oomycete plant parasitic *Phytophthora parasitica*, indicating that the cell death-inducing function might be related to the catalytic properties^46^. Here we showed that the active catalytic motifs are highly conserved in the tested sequences from various eukaryotes (Supplementary Fig. S1).

The observation that transcript for *HsPdi* was localized in oesophageal gland cells supports the hypothesis that the HsPDI protein is secreted into the host tissues to facilitate parasitism^8,49^. Furthermore, our expression analysis found that transcript abundance for *HsPdi* was increased significantly during early stages of infection reaching its maximum at 5 DAI, which coincides with rapid enlargement of the nematode induced syncytium^4^. This particular expression pattern points towards the importance of HsPDI in the early stages of infection including syncytium formation and maintenance. This hypothesis is further supported by results from infection assays where silencing *HsPdi* expression via RNAi led to impaired nematode development and ultrastructural and anatomical abnormalities in associated syncytium. Additionally, the described set of ultrastructural features strongly resembles the ultrastructure of syncytia associated with developing male juveniles^48^. This suggests that syncytia induced by *HsPdi* depleted-nematodes may suffer from a shortage of nutrients, which can lead to smaller females with a lower numbers of eggs. Earlier studies suggest that in *H. schachtii* (i) sex determination is regulated epigenetically by the composition and amount of nutrients withdrawn from syncytia^53^, (ii) sex differentiation occurs during the sedentary late J2 stage^3^. Therefore we conclude that *HsPdi* dsRNA-treated juveniles can induce fully functional syncytia that support their development into females during J2 sedentary stage. Afterwards these syncytia start to develop structural abnormalities. Theses abnormalities are similar to ultrastructural features of syncytium senescence typically occurring in degrading syncytia associated male J4 and adult males which had ceased feeding. The impaired function of their syncytia makes associated females developing smaller and producing fewer eggs.

It has been shown previously that ROS oxidize DNA, proteins, and lipids, which causes damage to the cellular organelles and inhibits cell functions^54^. Plant-parasitic nematodes encodes various antioxidant enzymes, such as superoxide dismutase (SOD), catalase, ascorbate, p-phenylenediamine-pyrocatechol (PPD-PC), o-dianisidine, guaiacol isoperoxidases, peroxiredoxins and glutathione peroxidases, which are important for parasitism and could have protective function against ROS^27,31,37,55^. The fact that *HsPdi* expression was elevated in presence of exogenous H_2_O_2_ and that silencing the expression of *HsPdi* using dsRNA decrease the tolerance of nematodes to 5 mM H_2_O_2_ points to the role of *HsPdi* to protect the nematode. These observations are supported by previous studies where a similar expression pattern was observed for *M incognita* peroxiredoxins. Silencing peroxiredoxins expression of *M incognita* impaired the nematode infectivity on tomato and their tolerance to exogenous H_2_O_2_^31^. We therefore conclude that *HsPdi* plays a dual role protecting nematodes against ROS and modulating the apoplastic redox status in the infected host tissue.

Our data for subcellular localization showed that HsPDI (without signal peptide) is localized in apoplast; however, this observation also raises the question of how an effector that is putatively secreted into the cytoplasm of infected tissues is ultimately translocated to the apoplastic space. Although the exact mechanism is currently unknown, it is plausible that host trafficking machinery is manipulated to deliver effectors into the apoplastic space^56,57^.

In plants, apoplastic ROS are actively produced through the action of NADPH oxidases and class III peroxidases, but the biological significance and the mechanism by which these ROS are scavenged during the plant–nematode interaction are not well understood. Recently, it was shown that *H. schachtii* infection of Arabidopsis plants stimulates ROS burst via host NADPH oxidases (RbohD and RbohF). Surprisingly, knocking out RbohD and RbohF restricted nematode development and nurse cell formation and triggered massive cell death upon nematode infection^25^. Combining the previous studies with our result we conclude that although ROS are needed for successful nematode infection, they are harmful for the plants and nematodes when exceeding a certain level. Therefore, nematode not only induce ROS burst, but they also manipulate their levels for optimal infection and less plant damage. HsPDI is proposed as one of the effectors which may serve this purpose. Alternatively, it is possible that Rbohs are guarded by a nucleotide-binding and leucine-rich repeat protein (NLR), leading to a strong immune response in mutants deficient in Rboh genes upon infection^9,58^.

In conclusion, our results strongly indicate that HsPDI is a nematode effector that is secreted into host tissues and become a part of the host antioxidant mechanisms as plant ROS scavenger not only during invasion but also during sedentary parasitism. Clarifying further details of the interplay between various ROS-producing and ROS-scavenging systems during plant-nematode interaction will provide exciting information on nematode parasitism.

## Methods

### Plant growth and nematode culture

*Arabidopsis thaliana* L. Heyn. ecotype Col-0 plants and two transgenic lines expressing HsPDI were grown aseptically on agar medium supplemented with modified 0.2 Knop’s nutrient solutions for 16 h light and 8 h dark at 25 °C as described previously^2^.

*Heterodera schachtii* Schmidt used in the experiments was reared on white mustard (*Sinapis alba* L. cv. Albatros) plants which were grown aseptically on agar containing 0.2% Knop medium. Mature cysts were collected in funnels and hatched in 3 mM ZnCl_2_^2^. The hatched pre-parasitic J2s were collected and used in the experiments.

### Infection assay

Nematode infection assays on Arabidopsis plants either for the nematode RNA interference (RNAi) experiments or on HsPDI-expressing lines were performed as described previously^25^. Briefly, sterilised seeds were placed on 0.2% Knop medium. After 10 days, roots were inoculated with 60–70 J2s per plant. For each experiment, 12 plants per treatment were used. Numbers of adult males and females were counted per plant at 12 days after inoculation (DAI). Furthermore, sizes of females and associated syncytia were measured after 13 DAI, sizes of cysts and numbers of eggs per cyst were examined at 45 DAI using Leica M165C Binocular (Leica Microsystems, Wetzlar, Germany) and Leica Application Suite software. Experiments were repeated three times and analysed using Student’s *t*-test.

### Sequence analysis

*HsPdi* (KU948160) was identified by performing a BLASTn of the esophageal gland cells putative secretory hsp3 (AF273730.1) which was isolated from *Heterodera glycines* ^50^ against draft transcriptomic data of *H. schachtii*. The draft transcriptome was generated by using next generation sequencing (Illumina, 100 bp paired end reads) of pre-parasitic J2s. The assembly was done using CLC genomics workbench after trimming the reads for adapter sequence and low quality nucleotides (less than 0.05) and ambiguity nucleotides (more than 2 adjacent ambiguous nucleotides).

The deduced protein was analysed to predict its functional domain(s). The conserved domains search was performed using the National Centre for Biotechnology Information NCBI CD search program (http://www.ncbi.nlm.nih.gov/Structure/cdd/wrpsb.cgi), signal peptide was identified using signalP4 server^59^, and transmembrane domains using TMHMM algorithm^60^.

The full PDI protein sequences from different organisms were obtained from NCBI (http://www.ncbi.nlm.nih.gov/), see Supplementary Table S1. All sequences were aligned using CLC Main Workbench (V7.7.3).

### *In situ* hybridization

Digoxigenin (DIG)-labelled probes complementary to identified *HsPdi* fragments were amplified in asymmetric PCR with single sense (negative control) or antisense primer and DIG-labelled deoxynucleoside triphosphates (dNTPs) (Roche) in the reaction mixture (see Supplementary Table S2). The hybridization was performed on the pre-parasitic J2s of *H. schachtii* at temperature of 47 °C as performed in previous study^61^. The hybridized nematodes were examined using Leica DMI2000 compound microscope.

### Developmental expression pattern analysis

Transcription of *HsPdi* was analysed in different developmental stages (eggs, pre-parasitic J2, parasitic juveniles and females) by quantitative PCR (qRT-PCR) using specific primers (Supplementary Table S2). Around 3,000 eggs and 3,000 pre-parasitic J2s per biological replicate were collected directly from hatching funnels^2^. Around 500–600 nematodes per biological replicate were collected manually from nematode infected roots of *A. thaliana* at 5, 10, 15 DAI, what corresponds to J3, J4, and young female stages.

Total RNA of the biological replicates was extracted using NucleoSpin RNA kit (MACHEREY-NAGEL) following manufacturer’s protocol. Quality and quantity of the extracted RNA samples were tested using the Agilent 2100 Bioanalyzer system (Agilent Technologies). The RNA with RNA integrity number (RIN) value higher than 8.5 was used for first strand cDNA synthesis using the High-Capacity cDNA Reverse Transcription Kit (Applied Biosystems) and oligo-dT primer. The resulted cDNAs were tested for the expression changes using the Stepone Plus Real-Time PCR System (Applied Biosystems) with 95 °C for 15 s and 60 °C for 30 s (40 cycles) for amplification. Each sample contained 10 μl of Fast SYBR Green qPCR Master Mix (Invitrogen), 9 μl of the specific primer mixture with final concentration 1 μM for each primer, 1 μl of cDNA. The amplified data were analysed by using the Stepone Plus Real-Time PCR software to create Ct values. The data were analysed and relative expression was calculated following Pfaffl^62^. Actin was used as internal control for all experiments (Supplementary Table S2). Three biological replicates from each stage in three technical replicates for each biological replicate were used.

### Double stranded RNA (dsRNA) and gene silencing in nematodes

*HsPdi* dsRNA was synthesized using RiboMAX RNA Large-Scale Production System (Promega) according to the manufacturer’s instruction. The forward and reverse primers (Supplementary Table S2) were supplemented with the SP6 and T7 promoter sequences and used for dsRNA synthesis. The *GFP* template was used for synthesis of a dsRNA construct that was used as a negative control.

About 10,000 freshly hatched J2s of *H. schachtii* were soaked in 50 µL of dsRNA incubation mixture consisting of 25 µL *HsPdi*-dsRNA or *GFP*-dsRNA (2 mg*mL^−1^) in 5 µL of 10x M9 buffer (55 mM KH_2_PO_4_, 21 mM NaCl, 47 mM NH_4_Cl) supplemented with 100 mM spermidine (1.5 µL), 500 mM octopamine (5.0 µL) and 13.5 µL (nematode suspension) for one day. Incubated J2s were washed three times in H_2_O, sterilized in 0.05 M HgCL_2_ for 2 minutes and washed again three times with sterile water. Afterwards, the batch of juveniles was divided equally and one part was used for plant infection assay whereas the second part was used to evaluate the level of *HsPdi* silencing using qRT-PCR as described above.

Ten days old *A. thaliana* Col-0 roots were inoculated with 60–70 J2s incubated in *HsPdi* or *GFP* dsRNA. Numbers of developed females and males were counted after 12 DAI, sizes of females and syncytia associated with females were measured at 13 DAI, whereas sizes of cysts and numbers of eggs per cyst were examined at 45 DAI. All measurements were conducted under a Leica M165C stereo microscope using manufacturer’s software. Experiments were repeated three times with 12 plants per treatment in each experiment. The obtained data were merged and analysed using the Student’s *t*-test.

### Syncytium anatomy and ultrastructure

Segments of roots containing nematode induced syncytia were dissected from Arabidopsis Col-0 plants inoculated with *HsPdi* or *GFP* dsRNA-treated juveniles at 5 and 10 DAI. They were processed for light and transmission electron microscopy as described^63^. Semi-thin sections (3 μm thick) taken on a Leica RM2165 microtome (Leica) were stained with hot 1% (w/v) aqueous solution of Crystal violet (Sigma, St. Louis, MI, USA) for 1 min at 65 °C. They were examined with an Olympus AX70 ‘Provis’ (Olympus, Tokyo, Japan) light microscope equipped with an Olympus DP50 digital camera. Ultra-thin sections (70–80 nm thick) taken on a Leica UCT ultramicrotome (Leica Microsystems) were collected and on formvar-coated (Fluka, Buchs, Switzerland) single-slot copper grids and stained with uranyl acetate (Fluka) and lead citrate (Sigma)^4^. They were examined with an FEI 268D ‘Morgagni’ transmission electron microscope (FEI, Hillsboro, OR, USA) operating at 80 kV. The images were recorded with an SIS ‘Morada’ digital camera (Olympus SIS, Münster, Germany) at 10 Mpix resolution. The images were equalized for similar contrast and brightness, resized and cropped using Adobe Photoshop graphic software.

### Survival of *H. schachtii* and *HsPdi* expression check under the H_2_O_2_ stress

Around 100–150 freshly hatched J2s were incubated in 0, 5, 10, 25 mM of H_2_O_2_. Dead nematodes were counted after 15, 30, 45 and 60 min and percentage of survival was calculated. Treatments were replicated three times and experiment was repeated three times. To check *HsPdi* gene expression under H_2_O_2_ stress, J2s were incubated for 30 min in 5 or 10 mM H_2_O_2_, and washed in sterile tap water. J2s incubated in sterile tap water were used as a control. RNA was extracted, cDNA was generated and expression of *HsPdi* was quantified by qRT-PCR as described above. Three biological replicates were carried out and each one was replicated three times.

### Effect of *HsPdi* depletion on nematode survival under the H_2_O_2_ stress

Around 150 freshly hatched J2s were incubated overnight in solution of *HsPdi* dsRNA and in *GFP* dsRNA as described above and then washed in tap water. Afterwards, the J2s were incubated in 5 mM H_2_O_2_ or in tap water as a control for 30 min. The numbers of dead J2s were counted and their percentage was calculated and analysed using Student’s *t*-test. Each treatment consisted of 4 replicates and the experiment was repeated 4 times.

### Construct generation and production of transgenic HsPDI expressing plants

The ORF of the *HsPdi* with no signal peptide was cloned into the binary Gateway over-expression vector pMDC83 to obtain a C-terminal fusion with GFP^64^ using the primers listed in Table S2. The construct was transferred to *Agrobacterium tumefaciens GV3101::pMP90* strain^65^ and transformed into *A. thaliana* Col-0 using the floral dip method^66^. The transformed plants were selected for hygromycin resistance on modified 0.2 Knop medium and grown for 3 generations to obtain homozygous lines for infection assays. The expression of *HsPdi* in homozygous lines was confirmed using qRT-PCR as described previously^62^.

The homozygous lines were grown on Murashige and Skoog media (MS) plates for 10 days. The number of lateral roots, main root length, fresh root weight and fresh shoot weight were measured and compared with Col-0. The experiment was repeated 3 times and each experiment consists of 10 plants for each line. After phenotyping, plants were infected with *H. schachtii* as described above.

### Agroinfiltration and subcellular localization of HsPDI

*A. tumefaciens* transformed with *HsPdi::pMDC83* construct was grown overnight in 50 ml of YEB liquid medium^65^ supplemented with 10 mg*mL^−1^ gentamycin, 50 mg*mL^−1^ kanamycin and 50 mg*mL^−1^ rifampicin to an OD600 of 0.8 in an incubator/shaker at 28 °C. Bacteria were pelleted then they were re-suspended in an infiltration buffer^65^. Bacterial suspensions were diluted with the infiltration buffer to get the required OD600 of 1. After incubation for 4 h at RT, the transformed bacteria were injected in the leaves abaxial side of 6 week-old *Nicotiana benthamiana* plant using 1 mL hypodermic syringe without needle. For co-infiltration of RNA silencing inhibitor P19 and the apoplastic marker, equal volumes of a bacterial suspensions harbouring *pBin61::P19* ^67^, *HsPdi::pMDC83* and apoplastic marker constructs were mixed and injected. To perform co-localization experiments in the apoplast, the N-terminal region of a membrane-localized receptor-like-kinase (At4g31250) was amplified and cloned in frame with mCherry under the control of the 35 S CaMV promoter and terminator. The cloning of only N-terminal region assured the delivery of fusion protein into extracellular region. The complete expression cassette was further cloned with restriction enzyme SdaI into the binary vector pGreenII^68^ resulting in a pGreen-apo-mCherry marker. The primers sequences are given in Table S2. Infiltrated plants were incubated in the growth chamber (16hrs light, 8hrs dark and at 25 °C) for 6 days. Slides were made from the infiltrated leaves and examined for the presence of fluorescence signal using Zeiss CLSM 710 with and without plasmolysis with 1 M NaCl_2_.

### ROS measurement in HsPDI over-expressing plants

ROS production in Col-0 and transgenic HsPDI over-expressing plants after treatment with bacterial peptide flg22 (100 nM) was measured on leaf disc samples using luminol-based assay and 96 wells plate luminometer Mithras LB 940 (Berthold Technologies) as described previously^69^. Light emission was measured in relative units over 120 min long incubation period and analysed using instrument software. ROS production was checked in transgenic plants expressing the nematode Hs-Tyr^70^ and compared with Col-0 as a control. Data was tabulated and analysed statistically using Student’s *t*-test. Four leaf discs were used per line as technical replicates. The experiment was repeated three times. Total ROS production was calculated and presented.

## Electronic supplementary material

Supplementary Information

## References

[1] Wyss U, Zunke U (1986). Observations on the behaviour of second stage juveniles of *Heterodera schachtii* inside host roots. Rev. Nematol..

[2] Sijmons PC, Grundler FMW, Mende N, von, Burrows PR, Wyss U (1991). *Arabidopsis thaliana* as a new model host for plant-parasitic nematodes. Plant J..

[3] Wyss U, Grundler FMW (1992). Feeding-Behavior of Sedentary Plant Parasitic Nematodes. Netherlands J. Plant Pathol..

[4] Golinowski W, Grundler FMW, Sobczak M (1996). Changes in the structure of *Arabidopsis thaliana* during female development of the plant-parasitic nematode *Heterodera schachtii*. Protoplasma.

[5] Szakasits D (2009). The transcriptome of syncytia induced by the cyst nematode *Heterodera schachtii* in Arabidopsis roots. J. Exp. Bot..

[6] Hofmann J (2010). Metabolic profiling reveals local and systemic responses of host plants to nematode parasitism. Plant J..

[7] Williamson VM, Gleason CA (2003). Plant-nematode interactions. Curr. Opin. Plant Biol..

[8] Mitchum MG (2013). Nematode effector proteins: An emerging paradigm of parasitism. New Phytol.

[9] Holbein J, Grundler FM, Siddique S (2016). Plant basal resistance to nematodes: an update. J. Exp. Bot..

[10] Patel N (2010). A nematode effector protein similar to annexins in host plants. J. Exp. Bot..

[11] Hewezi T (2010). Arabidopsis spermidine synthase is targeted by an effector protein of the cyst nematode *Heterodera schachtii*. Plant Physiol..

[12] Siddique S (2015). A parasitic nematode releases cytokinin that controls cell division and orchestrates feeding site formation in host plants. Proc. Natl. Acad. Sci..

[13] Wang J (2011). Identification of potential host plant mimics of CLAVATA3/ESR (CLE)-like peptides from the plant-parasitic nematode *Heterodera schachtii*. Mol. Plant Pathol..

[14] Mittler R, Vanderauwera S, Gollery M, Van Breusegem F (2004). Reactive oxygen gene network of plants. Trends Plant Sci..

[15] Torres MA, Jones JDG, Dangl JL (2006). Reactive Oxygen Species Signalling in Response to Pathogens. Plant Physiol..

[16] Quentin M, Abad P, Favery B (2013). Plant parasitic nematode effectors target host defense and nuclear functions to establish feeding cells. Front Plant Sci.

[17] Goverse A, Smant G (2014). The activation and suppression of plant innate immunity by parasitic nematodes. Annu Rev Phytopathol.

[18] Shetty NP (2007). Role of hydrogen peroxide during the interaction between the hemibiotrophic fungal pathogen *Septoria tritici* and wheat. New Phytol.

[19] Slesak I, Libik M, Karpinska B, Karpinski S, Miszalski Z (2007). The role of hydrogen peroxide in regulation of plant metabolism and cellular signalling in response to environmental stresses. Acta Biochim. Pol..

[20] Veal EA, Day AM, Morgan BA (2007). Hydrogen peroxide sensing and signalling. Mol. Cell.

[21] Forman HJ, Maiorino M, Ursini F (2010). Signaling functions of reactive oxygen species. Biochemistry.

[22] Torres MA, Dangl JL, Jones JD (2002). Arabidopsis gp91phox homologues AtrbohD and AtrbohF are required for accumulation of reactive oxygen intermediates in the plant defense response. Proc. Natl. Acad. Sci..

[23] Melillo MT, Leonetti P, Bongiovanni M, Castagnone-Sereno P, Bleve-Zacheo T (2006). Modulation of reactive oxygen species activities and H2O2 accumulation during compatible and incompatible tomato-root-knot nematode interactions. New Phytol.

[24] Waetzig G, Grundler F, Sobczak M (1999). Localization of hydrogen peroxide during the defence response of *Arabidopsis thaliana* against the plant-parasitic nematode *Heterodera glycines*. Nematol..

[25] Siddique S (2014). Parasitic worms stimulate host NADPH oxidases to produce reactive oxygen species that limit plant cell death and promote infection. Science Signal..

[26] Foley RC, Gleason CA, Anderson JP, Hamann T, Singh KB (2013). Genetic and genomic analysis of *Rhizoctonia solani* interactions with Arabidopsis; Evidence of resistance mediated through NADPH oxidases. PLoS One.

[27] Jones JT, Reavy B, Smant G, Prior AE (2004). Glutathione peroxidases of the potato cyst nematode *Globodera rostochiensis*. Gene.

[28] Molina L, Kahmann R (2007). An *Ustilago maydis* gene involved in H_2_O_2_ detoxification is required for virulence. Plant Cell.

[29] Blackman LM, Hardham R (2008). Regulation of catalase activity and gene expression during *Phytophthora nicotianae* development and infection of tobacco. Mol. Plant Pathol..

[30] Flores-Cruz Z, Allen C (2009). *Ralstonia solanacearum* encounters an oxidative environment during tomato infection. Mol. Plant Microbe Interact..

[31] Dubreuil G (2011). Peroxiredoxins from the plant parasitic root-knot nematode, *Meloidogyne incognita*, are required for successful development within the host. Int. J. Parasitol.

[32] Li Z, Liu X, Chu Y, Wang Y, Zhang Q, Zhou X (2011). Cloning and characterization of a 2-Cys peroxiredoxin in the pine wood nematode, *Bursaphelenchus xylophilus*, a putative genetic factor facilitating the infestation. Int J Biol Sci.

[33] Henkle-Dührsen K, Kampkötter A (2001). Antioxidant enzyme families in parasitic nematodes. Mol Biochem Parasitol.

[34] Sotirchos IM, Hudson AL, Ellis J, Davey MW (2009). A unique thioredoxin of the parasitic nematode *Haemonchus contortus* with glutaredoxin activity. Free Radic Biol Med.

[35] Kunchithapautham K, Padmavathi B, Narayanan RB, Kaliraj P, Scott AL (2003). Thioredoxin from *Brugia malayi*: Defining a 16-kiloDalton class of thioredoxins from nematodes. Infect. Immun..

[36] Lin B (2016). A novel nematode effector suppresses plant immunity by activating host reactive oxygen species-scavenging system. New Phytol.

[37] Robertson L (2000). Cloning, expression and functional characterisation of a peroxiredoxin from the potato cyst nematode *Globodera rostochiensis*. Mol. Biochem. Parasitol..

[38] Appenzeller-Herzog C, Ellgaard L (2008). The human PDI family: versatility packed into a single fold. Biochimica et Biophysica Acta.

[39] Ali Khan H, Mutus B (2014). Protein disulfide isomerase a multifunctional protein with multiple physiological roles. Frontiers in Chemistry.

[40] Frand AR, Kaiser CA (1999). Ero1p oxidizes protein disulfide isomerase in a pathway for disulfide bond formation in the endoplasmic reticulum. Mol. Cell.

[41] De APAM (2011). Protein disulfide isomerase redox-dependent association withp47(phox): evidence for an organizer role in leukocyte NADPH oxidase activation. J. Leukoc. Biol..

[42] Stolf BS (2011). Protein disulfide isomerase and host-pathogen interaction. Scientific World J..

[43] Ben Achour Y, Chenik M, Louzir H, Dellagi K (2002). Identification of a disulfide isomerase protein of *Leishmania* major as a putative virulence factor. Infect. Immun..

[44] Hong BX, Soong L (2008). Identification and enzymatic activities of four protein disulfide isomerase (PDI) isoforms of *Leishmania amazonensis*. Parasitol. Res..

[45] Mahajan B (2006). Protein disulfide isomerase assisted protein folding in malaria parasites. Int. J. Parasitol.

[46] Meng Y (2015). The protein disulfide isomerase 1 of *Phytophthora parasitica* (PpPDI1) is associated with the haustoria-like structures and contributes to plant infection. Front Plant Sci.

[47] Kozlov G, Maattanen P, Thomas DY, Gehring K (2010). A structural overview of the PDI family of proteins. ‎FEBS J..

[48] Sobczak M, Golinowski W, Grundler FMW (1997). Changes in the structure of *Arabidopsis thaliana* roots induced during development of males of the plant parasitic nematode *Heterodera schachtii*. Eur. J. Plant Pathol..

[49] Maier TR, Hewezi T, Peng J, Baum TJ (2013). Isolation of whole esophageal gland cells from plant-parasitic nematodes for transcriptome analyses and effector identification. Mol Plant Microbe Interact.

[50] Wang X (2001). Signal peptide-selection of cDNA cloned directly from the esophageal gland cells of the soybean cyst nematode *Heterodera glycines*. Mol. Plant Microbe Interact..

[51] Jones JT (2009). Identification and functional characterization of effectors in expressed sequence tags from various life cycle stages of the potato cyst nematode *Globodera pallida*. Mol Plant Pathol.

[52] Kim J, Mayfield SP (2002). The active site of the thioredoxin-like domain of chloroplast protein disulfide isomerase, RB60, catalyzes the redox-regulated binding of chloroplast poly(A)-binding protein, RB47, to the 5′ untranslated region of psbA mRNA. Plant Cell Physiol..

[53] Müller J, Rehbock K, Wyss U (1982). Growth of *Heterodera schachtii* with remarks on amounts of food consumed. Revue de Nématologie.

[54] Baker CJ, Orlandi EW (1995). Active oxygen in plant pathogenesis. Annu. Rev. Phytopathol.

[55] Molinari S, Miacola C (1997). Antioxidant enzymes in phytoparasitic nematodes. J. Nematol..

[56] Wang J (2010). Dual roles for the variable domain in protein trafficking and host-specific recognition of *Heterodera glycines* CLE effector proteins. New Phytol.

[57] Ali S (2015). Analysis of putative apoplastic effectors from the nematode, *Globodera rostochiensis*, and identification of an expansin-like protein that can induce and suppress host defences. PLoS One.

[58] Kadota Y (2014). Direct regulation of the NADPH oxidase RBOHD by the PRR-associated kinase BIK1 during plant immunity. Mol. Cell.

[59] Petersen TN, Brunak S, Heijne Gvon, Nielsen H (2011). SignalP 4.0: discriminating signal peptides from transmembrane regions. Nat. Methods.

[60] Krogh A, Larsson B, Heijne Gvon, Sonnhammer EL (2001). Predicting transmembrane protein topology with a hidden markov model: application to complete genomes. J. Mol. Biol..

[61] de Boer JM, Yan Y, Smant G, Davis EL, Baum TJ (1998). *In-situ* Hybridization to Messenger RNA in *Heterodera glycines*. J. Nematol..

[62] Pfaffl MW (2001). A new mathematical model for relative quantification in real-time RT-PCR. Nucleic Acids Res..

[63] Daneshkhah R (2013). *Piriformospora indica* antagonizes cyst nematode infection and development in Arabidopsis roots. J. Exp. Bot..

[64] Curtis MD, Grossniklaus U (2003). A gateway cloning vector set for high-throughput functional analysis of genes in planta. Plant Physio..

[65] Sparkes IA, Runions J, Kearns A, Hawes C (2006). Rapid, transient expression of fluorescent fusion proteins in tobacco plants and generation of stably transformed plants. Nat. Protoc..

[66] Clough SJ, Bent AF (1998). Floral dip: A simplified method for Agrobacterium-mediated transformation of *Arabidopsis thaliana*. Plant J..

[67] Voinnet O, Rivas S, Mestre P, Baulcombe D (2003). An enhanced transient expression system in plants based on suppression of gene silencing by the p19 protein of tomato bushy stunt virus. Plant J..

[68] Hellens RP, Edwards EA, Leyland NR, Bean S, Mullineaux PM (2000). pGreen: a versatile and flexible binary Ti vector for Agrobacterium-mediated plant transformation. Plant Mol. Biol..

[69] Prince DC, Drurey C, Zipfel C, Hogenhout S (2014). The leucine-rich repeat receptor-like kinase brassinosteroid insensitive1-associated kinase1 and the cytochrome P450 phytoalexin deficient3 contribute to innate immunity to aphids in Arabidopsis. Plant Physiol..

[70] Habash, S. S. *et al*. *Heterodera schachtii* Tyrosinase-like protein - a novel nematode effector modulating plant hormone homeostasis. *Sci. Rep*. **7**, 10.1038/s41598-017-07269-7 (2017). 10.1038/s41598-017-07269-7 PMC5537230 28761178

