# Supplementary material for: Identification and characterization of a putative protein disulfide isomerase (HsPDI) as an alleged effector of Heterodera schachtii
Source: Sci Rep. 2017 Oct 19;7:13536. doi: 10.1038/s41598-017-13418-9 (PMC5648851; doi:10.1038/s41598-017-13418-9)
Supplement: Supplementary file 1 — Supplementary Information [file 41598_2017_13418_MOESM1_ESM.doc]

### Identification and characterization of a putative protein disulfide isomerase (HsPDI) as an alleged effector of *Heterodera schachtii*

Samer S. Habash^1^, Miroslaw Sobczak^2^, Shahid Siddique^1^, Boris Voigt^3^, Abdelnaser Elashry^1,4^, Florian M.W. Grundler^1^

^1^Rheinische Friedrich-Wilhelms-University of Bonn, INRES – Molecular Phytomedicine, Karlrobert-Kreiten-Straße 13, D-53115 Bonn, Germany

^2^Department of Botany, Warsaw University of Life Sciences (SGGW), Nowoursynowska 159, PL-02787 Warsaw, Poland

^3^Rheinische Friedrich-Wilhelms-University of Bonn, Department of Plant Cell Biology, Institute of Cellular and Molecular Botany, Kirschallee 1, D-53115 Bonn, Germany

^4^ current address: Strube Research GmbH & Co. KG, Hauptstraße 1, 38387 Söllingen , Germany

Author for correspondence:

Florian M.W. Grundler

Tel: 00490228731675

E-mail: [grundler@uni-bonn.de](mailto:grundler@uni-bonn.de)

**Supplementary information**

**
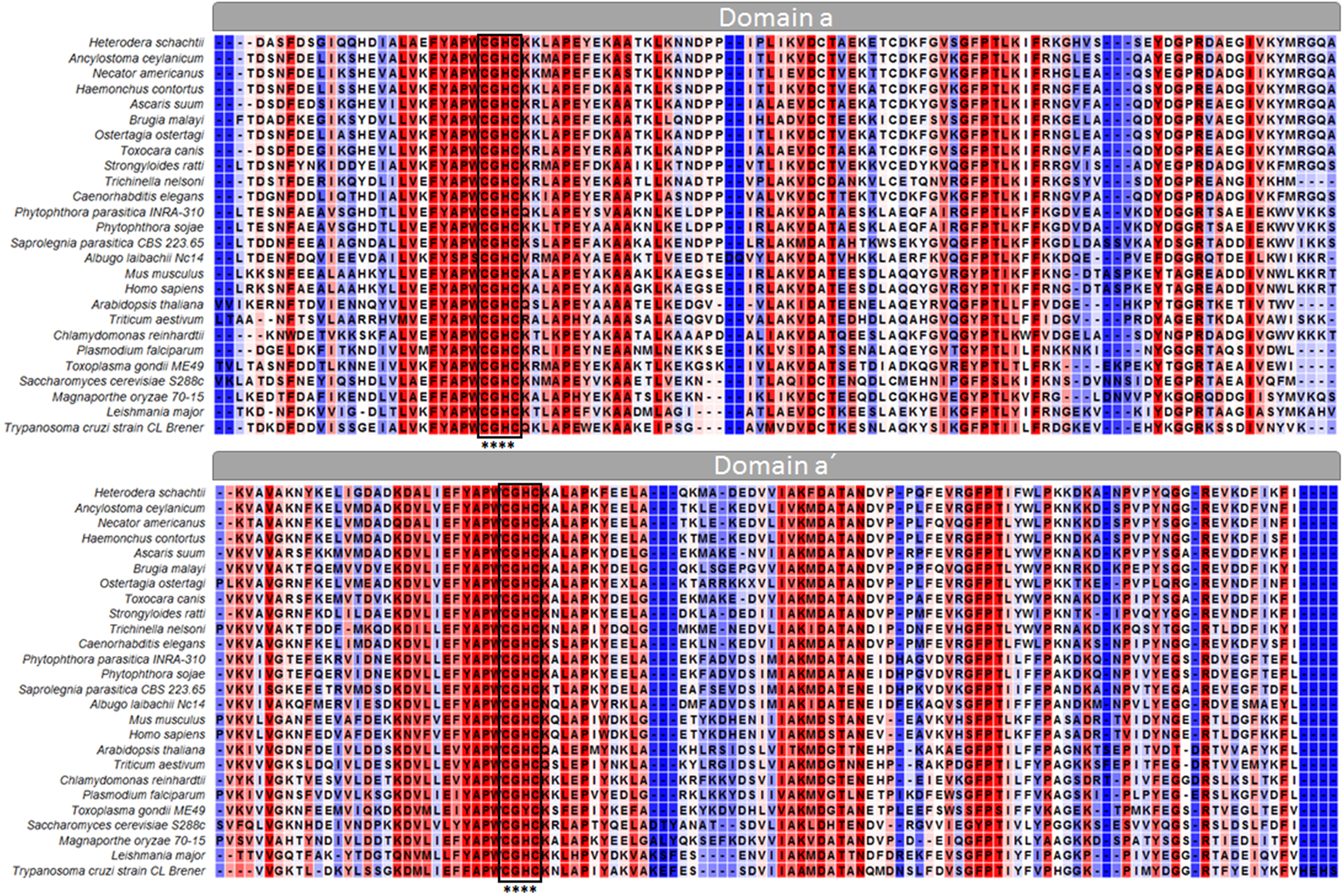
**

**Supplementary Fig. S1 Multiple sequence alignment of the a and a´ thioredoxin damains from HsPDI and PDIs from other organisms**. The regions with red colour background show the conserved sequences with high similarity. The blue coloured background represent the non-conserved sequences. The sequence in the black box shows the active catalytic motifs (CGHC). Alignment was performed using CLC Main Workbench (V7.7.3).


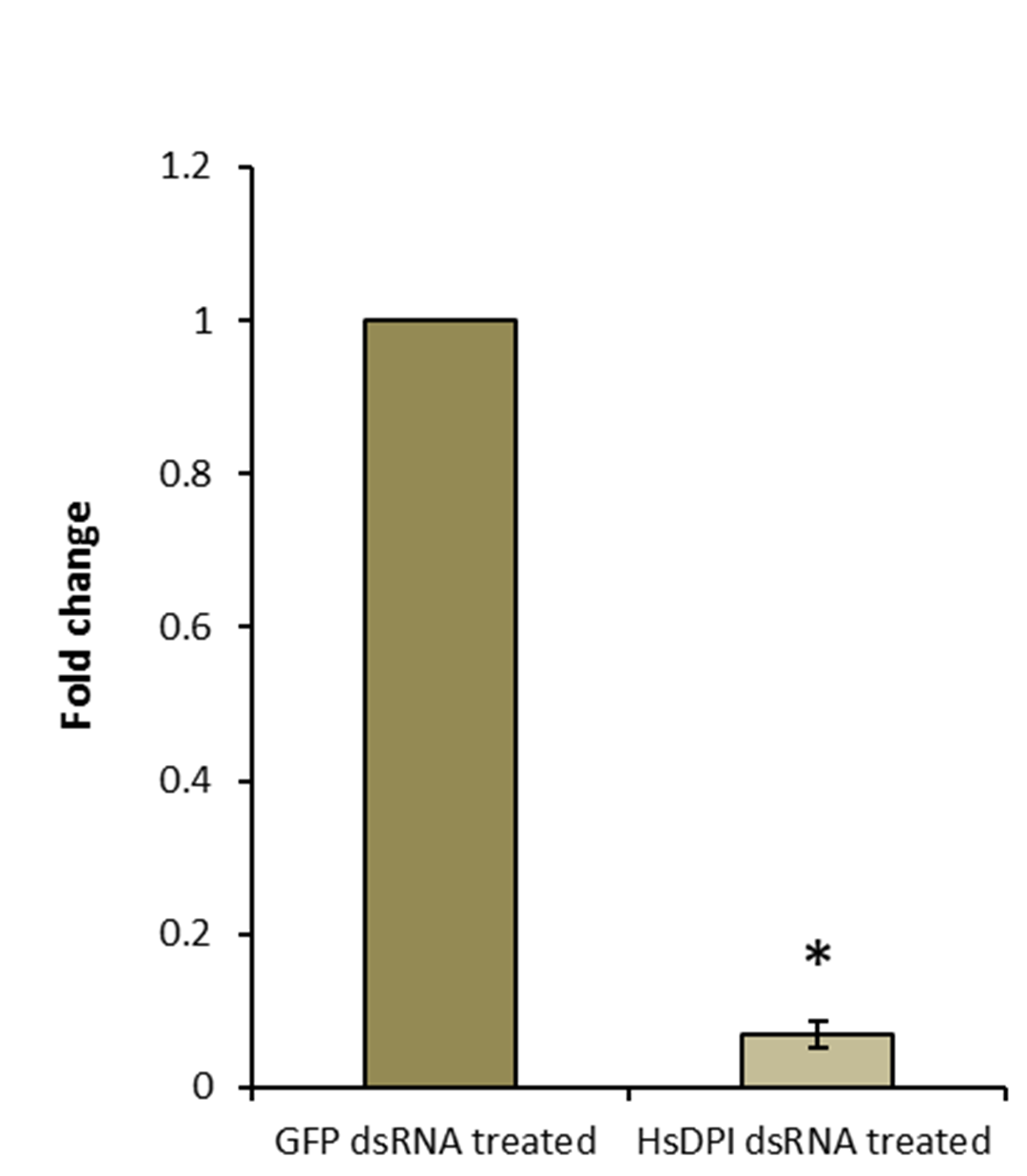


**Supplementary Fig. S2 *HsPdi* expression in nematode after treatment with dsRNA.** Freshly hatched J2s were incubated in *HsPdi* dsRNA or GFP dsRNA as negative control. Data are based on three independent experiments. Each bar represents the mean ± standard error of n =9. Asterisk marks indicates significant differences based on Student's *t*-test (P < 0.05).


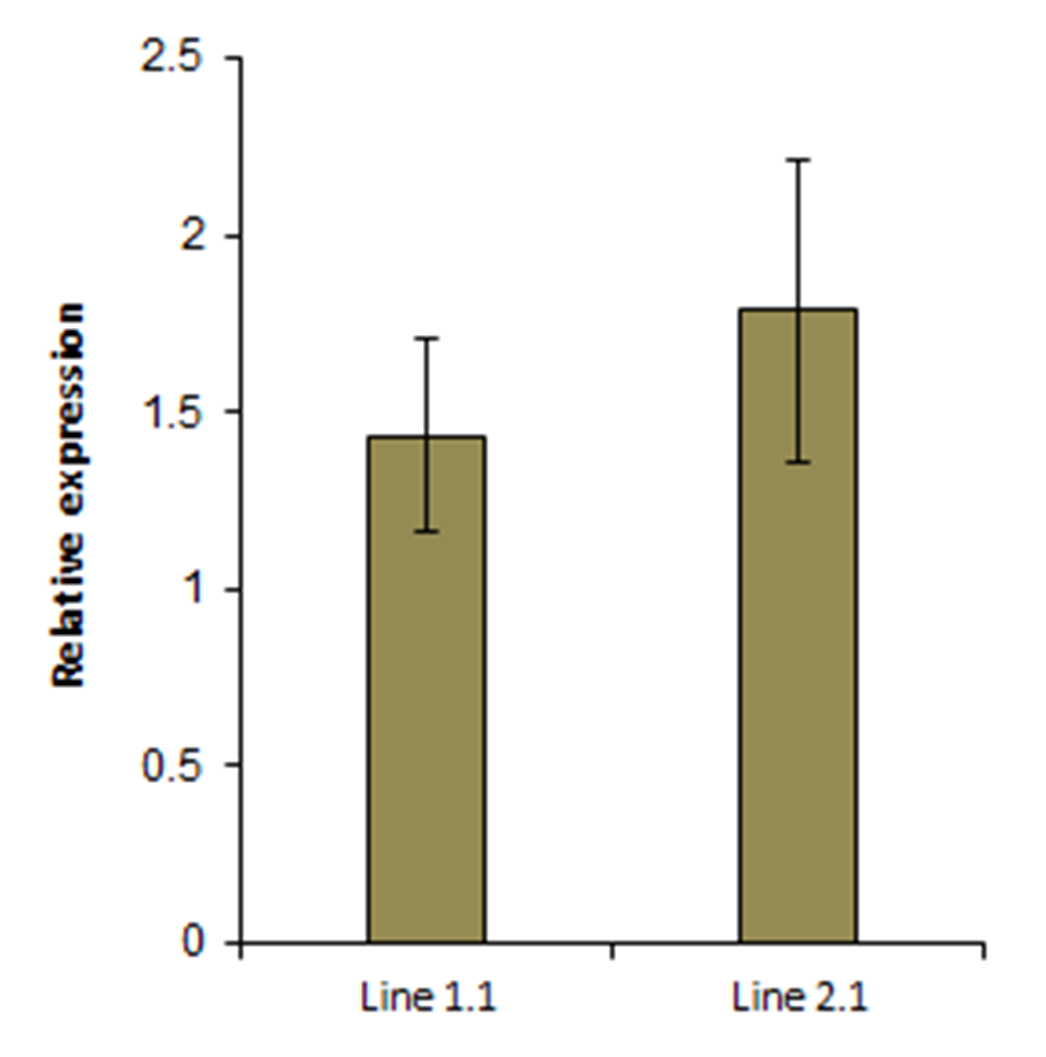


**Supplementary Fig. S3 Expression of *HsPdi* transcripts in transgenic Arabidopsis confirmed by qRT-PCR.** The qRT-PCR was performed on two independent T3, homozygous Arabidopsis lines. The expression of the transgene was determined in relation to the Arabidopsis housekeeping gene Actin. Each bar represents the mean ± standard error of n =9. Asterisk marks indicates significant differences based on Student's *t*-test (P < 0.05).


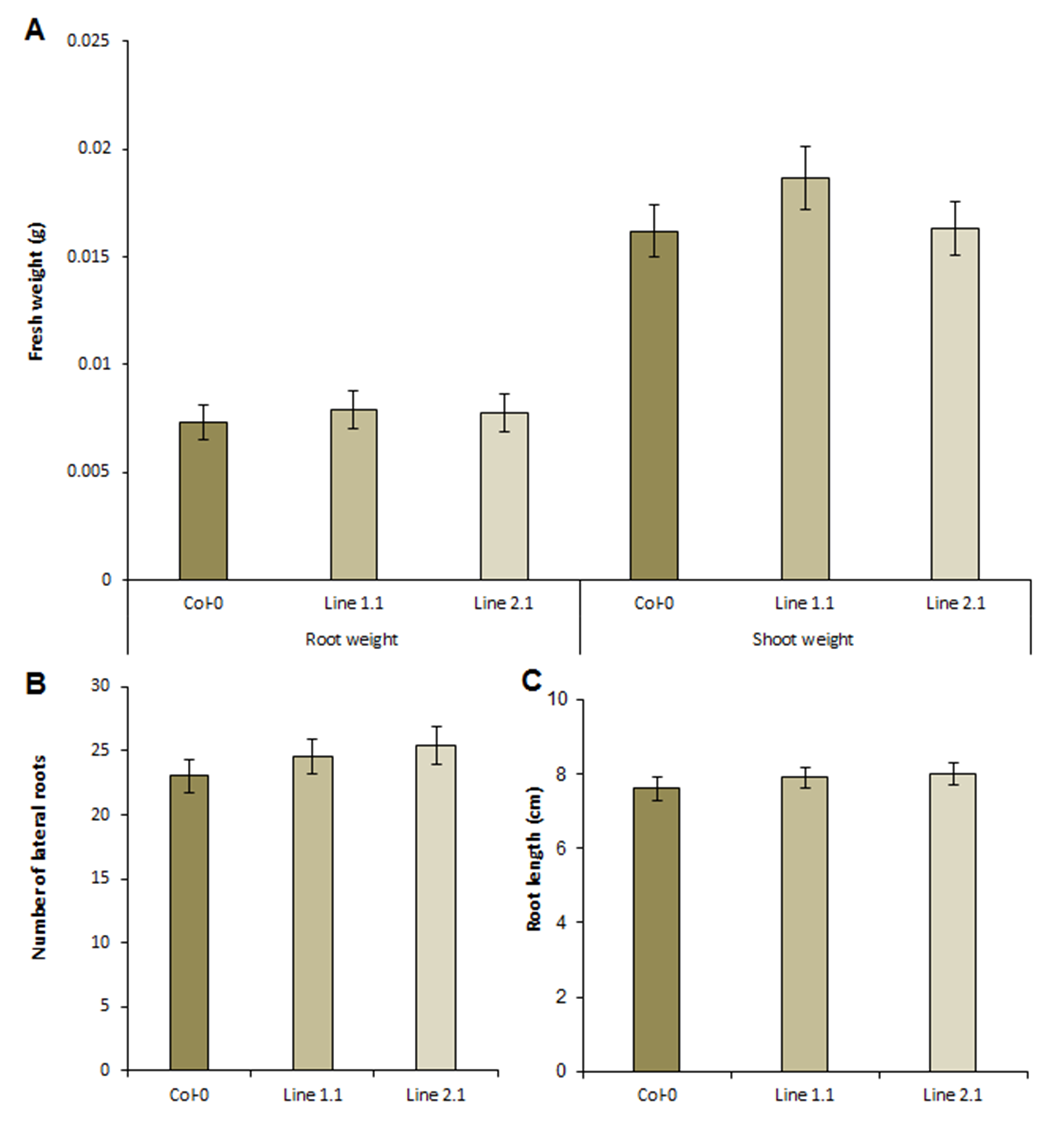


**Supplementary Fig. S4 Effect of ectopic expression of HsPDI on Arabidopsis growth.** The following parameters were analysed: (A) root and shoot fresh weight (B) number of lateral roots per plant (C) length of main root compared with Col-0. Data are based on three independent experiments. Each bar represents the mean ± standard error of n = 30. Asterisk marks indicates significant differences based on Student's *t*-test (P < 0.05).


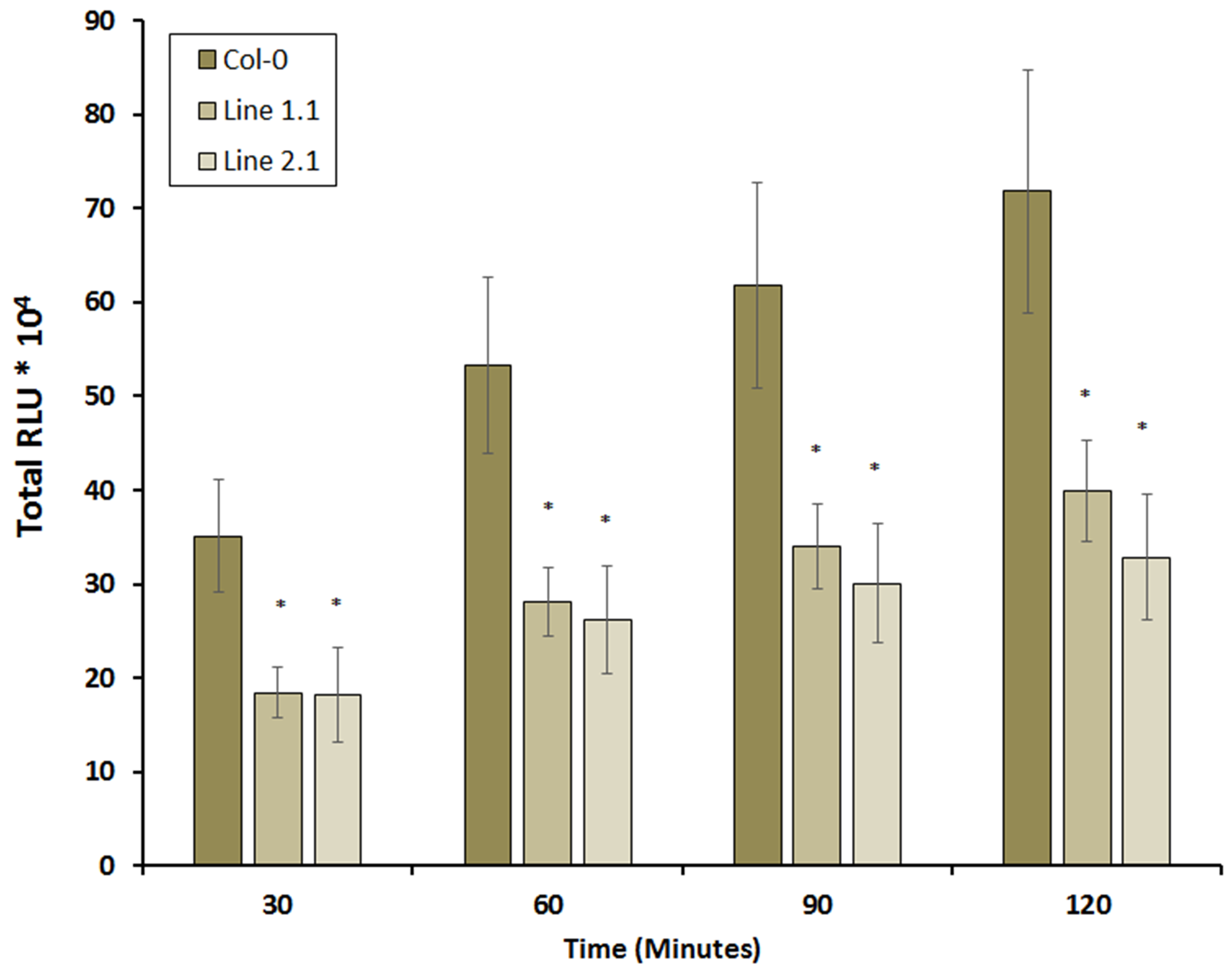


**Supplementary Fig. S5** **ROS bursts in response to the bacterial elicitor peptide flg22 in transgenic lines.** ROS burst was measured in relative light units (RLU) in plants expressing HsPDI and compared with Col-0 using luminol-based assay after 30, 60, 90 and 120 min-long incubation. Data are based on three independent experiments. Each bar represents the mean ± standard error of n = 12. Asterisk marks indicates significant differences based on Student's *t*-test (P < 0.05).


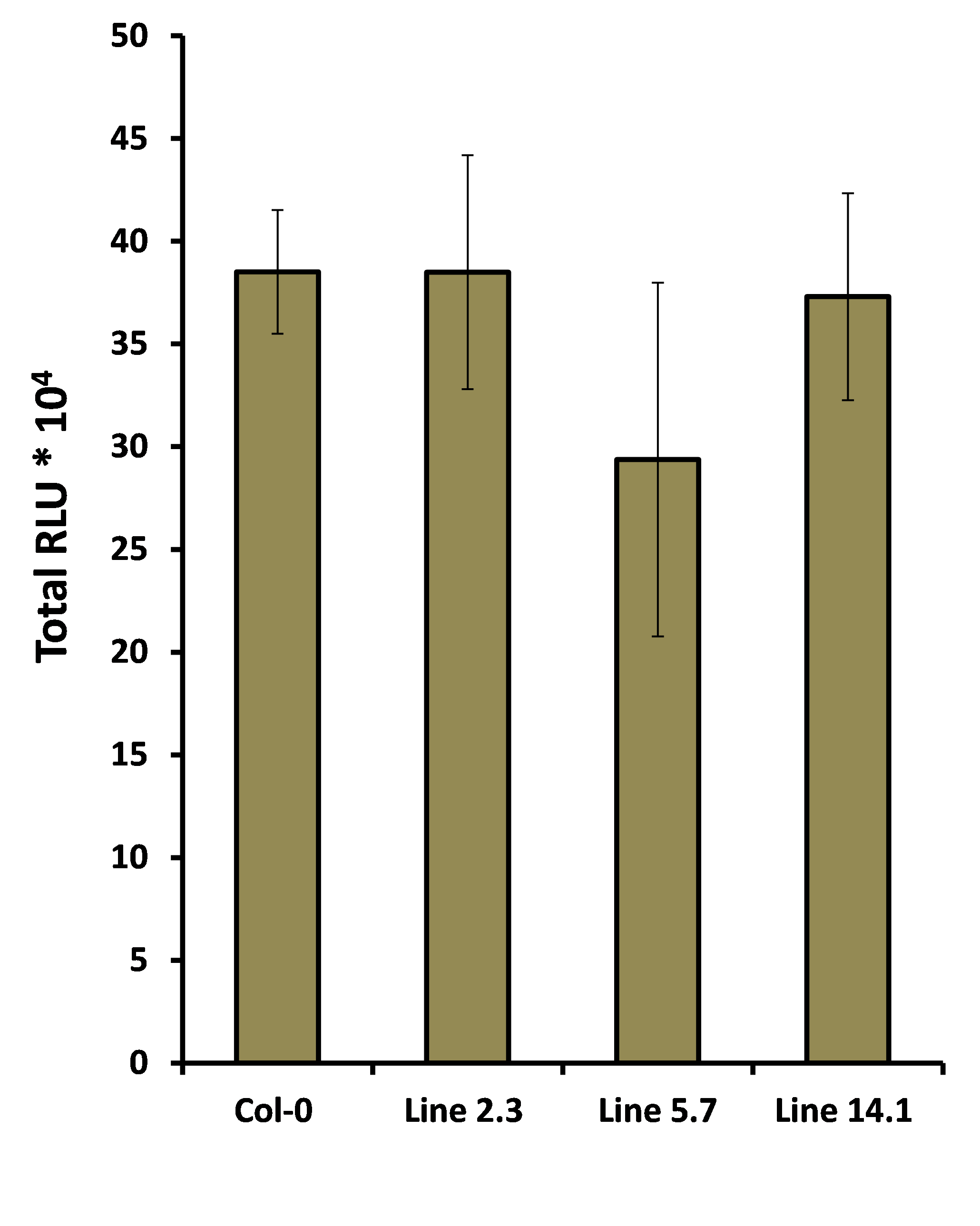


**Supplementary Fig. S6** **ROS burst in response to the bacterial elicitor peptide flg22 in Hs-Tyr transgenic lines.** ROS burst was measured in relative light units (RLU) in plants expressing Hs-Tyr and compared with Col-0 using luminol-based assay after 120 min-long incubation. Data are based on three independent experiments. Each bar represents the mean ± standard error of n = 12. Asterisk marks indicates significant differences based on Student's *t*-test (P < 0.05).


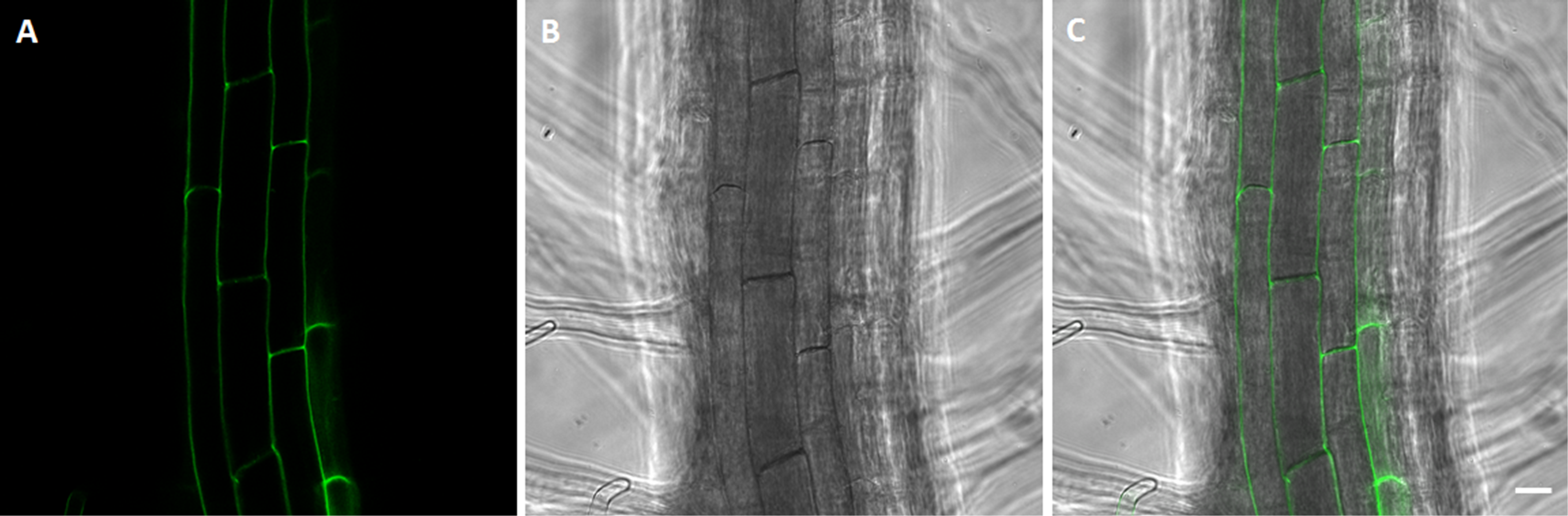


**Supplementary Fig. S7** Subcellular localization of HsPDI:GFP within transgenic *Arabidopsis thaliana* roots. Florescent signal of HsPDI::GFP was observed under confocal microscope in the apoplast of the root cells. (A) Dark filed (B) bright field (C) Merged photo. Bars = 10μm.

**Supplementary Table S1 Details of sequences used in the study.**

| **Species** | **Contig name/ Accession number** | **Identity %** |
| --- | --- | --- |
| *Ancylostoma ceylanicum* | EYC45980 | 76.6 |
| *Necator americanus* | XP_013298836 | 74.8 |
| *Haemonchus contortus* | CDJ86767 | 76.5 |
| *Ascaris suum* | ERG84937 | 71.3 |
| *Brugia malayi* | XP_001897232 | 36.9 |
| *Ostertagia ostertagi* | CAD29445 | 71.5 |
| *Toxocara canis* | KHN78570 | 72.9 |
| *Strongyloides ratti* | CEF66381 | 70.5 |
| *Trichinella nelsoni* | KRX16041 | 56.8 |
| *Caenorhabditis elegans* | NP_491995 | 73.3 |
| *Phytophthora parasitica* | XP_008914616 | 36.9 |
| *Phytophthora sojae* | XP_009520350 | 36.9 |
| *Saprolegnia parasitica* | KDO30563 | 37.7 |
| *Albugo laibachii* | CCA26649 | 32.8 |
| *Mus musculus* | NP_035162 | 33.8 |
| *Homo sapiens* | NP_000909 | 34 |
| *Arabidopsis thaliana* | NP_851234 | 31 |
| *Triticum aestivum* | BAO79451 | 31.7 |
| *Chlamydomonas reinhardtii* | XP_001701755 | 33.3 |
| *Plasmodium falciparum* | CAC15387 | 30.2 |
| *Toxoplasma gondii* | XP_002371293 | 32.3 |
| *Saccharomyces cerevisiae* | NP_009887 | 31.9 |
| *Magnaporthe oryzae* | XP_003710672 | 37.8 |
| *Leishmania major* | AAN75008 | 28.9 |
| *Trypanosoma cruzi* | XP_821173 | 31 |

**Supplementary Table S2 Primer labels and sequences used in the study**

| **Primer label** | **Primer sequence** |
| --- | --- |
| *HsPdi*-In situ-F | GAAGGAGAAAGCAAGCTG |
| *HsPdi* -In situ-R | TGCACTTTGCGCTTGTAA |
| *HsPdi* -qRT-PCR- F | CGAACAATCCACCGACCCTC |
| *HsPdi* -qRT-PCR- R | ACATTAGGGGAGAAGGAG |
| Hs-Actin-F | CGTGACCTCACTGACTACCT |
| Hs-Actin-R | CGTAGCACAACTTCTCCTTG |
| RNAi-F | TAATACGACTCACTATAGGGAGA GAAGGAGAAAGCAAGCTG |
| RNAi-R | CATACGATTTAGGTGACACTATAG TGCACTTTGCGCTTGTAA |
| *HsPdi* Localization Fw | CTAGCGATGTGTTGGAATA |
| *HsPdi* Localization Rw | GAGTTCCTCAGCCTTTGC |
| At-Actin-F | ACAGCAGAGCGGGAAATTGT |
| At-Actin-R | AGCAGCTTCCATTCCCACAA |
| Rlk Fw | gcCCATGGCAATGACCCGTGATGACAAATTC |
| RlkNter Rw | gcCCATGGGCGGACGAGTGTATCTGCACGG |
